# TflosYOLO+TFSC: an accurate and robust model for estimating flower count and flowering period

**DOI:** 10.3389/fpls.2025.1690413

**Published:** 2025-11-14

**Authors:** Qianxi Mi, Pengcheng Yuan, Chunlei Ma, Jiedan Chen, Mingzhe Yao

**Affiliations:** 1 Key Laboratory of Biology, Genetics and Breeding of Special Economic Animals and Plants, Ministry of Agriculture and Rural Affairs, Tea Research Institute of the Chinese Academy of Agricultural Sciences, Hangzhou, China; 2 National Key Laboratory for Tea Plant Germplasm Innovation and Resource Utilization, Tea Research Institute of the Chinese Academy of Agricultural Sciences, Hangzhou, China

**Keywords:** precision horticulture, deep learning, tea flower, flowering quantifying, computer vision

## Abstract

Tea flowers play a crucial role in taxonomic research and hybrid breeding of tea plants. As traditional methods of observing tea flower traits are labor-intensive and inaccurate, TflosYOLO and Tea Flowering Stage Classification (TFSC) models were proposed for tea flowering quantification, which enable the estimation of flower count and flowering period. In this study, a highly representative and diverse dataset was constructed by collecting flower images from 29 tea accessions in 2 years. Based on this dataset, the TflosYOLO model was built on the YOLOv5 architecture and enhanced with the Squeeze-and-Excitation (SE) network, Adaptive Rectangular Convolution, and Attention Free Transformer, which is the first model to offer a viable solution for detecting and counting tea flowers. The TflosYOLO model achieved a mean Average Precision at 50% IoU (mAP50) of 0.844, outperforming YOLOv5, YOLOv7, and YOLOv8. Furthermore, the TflosYOLO model was tested on 31 datasets encompassing 26 tea accessions and five flowering stages, demonstrating high generalization and robustness. The correlation coefficient (R^2^) between the predicted and actual flower counts was 0.964. Additionally, the TFSC model—a seven-layer neural network—was designed for the automatic classification of the flowering period. The TFSC model was evaluated for 2 years and achieved an accuracy of 0.738 and 0.899. Using the TflosYOLO+TFSC model, the tea flowering dynamics were monitored, and the changes in flowering stages were tracked across various tea accessions. The framework provides crucial support for tea plant breeding programs and the phenotypic analysis of germplasm resources.

## 1 Introduction

Tea is one of the three major beverages in the world, and the tea plant is an important economic crop in multiple countries. With a cultivation history spanning thousands of years, China is home to a rich diversity of native tea accessions. In recent years, numerous distinct tea cultivars have been developed across various tea-growing regions, supporting the growth of the tea industry and promoting improvements in both quality and efficiency. As a perennial leaf crop, the economic value of the tea plant primarily derives from its young shoots, and most research has focused on the growth and development of these shoots. However, as reproductive organs, tea flowers are crucial for conducting genetic and taxonomic studies. The flowering period is crucial for selecting parent plants for hybrid breeding, as it must be relatively synchronized for successful cross-breeding. Tea flowering consumes the plant nutrients so that flower thinning can regulate carbon–nitrogen metabolism, promoting vegetative growth while suppressing reproductive growth, further enhancing the yield of young shoots and increasing the amino acid content, which positively impacts tea quality (Tan et al., 2024). Therefore, measuring the floral phenotypes of tea accessions is of great importance.

China has abundant phenotypic resources of tea plants, and significant differences exist between accessions in terms of flower quantity and flowering stage (including the onset and cessation of blooming, and the duration of the flowering stage). Breeding programs require investigations of flower quantity and flowering stages. However, traditional methods for observing tea flower traits, such as manual measurements, are labor-intensive and prone to inaccuracies. Additionally, previous studies have only selected a small number of accessions, making it difficult to accurately describe the regional characteristics of the species. Therefore, there is a clear need to develop efficient, precise, and highly generalized phenotyping technologies for tea flowers.

In recent years, advancements in machine learning, deep learning, computer vision technologies, and drones have significantly impacted agricultural applications, such as yield prediction, crop growth monitoring, automated harvesting, and quality detection. Traditional machine learning (ML) methods, including support vector machine (SVM), random forest, partial least squares regression (PLSR), K-means clustering, and artificial neural network (ANN), take a data-driven approach to model the relationships between input data and labels, such as crop yield (Paudel et al., 2021). These machine learning systems are capable of processing large datasets and handling non-linear tasks efficiently (Chlingaryan et al., 2018). For example, a machine learning algorithm incorporating K-means clustering was developed for grapevine inflorescence detection, classification, and flower number estimation, which demonstrated high accuracy (Liu et al., 2018). In another study, six different machine learning algorithms, including ridge regression, SVM, random forest, Gaussian process, K-means, and Cubist, were utilized by Song et al. (2023) to establish yield prediction models based on drone-collected visible and multispectral images of wheat canopies during the grain filling stage. As for machine learning in tea research, Tu et al. (2018) utilized Unmanned Aerial Vehicle (UAV)-acquired hyperspectral data to build a classification model for tea accessions and estimate the content of key chemicals related to tea flavor. Their research indicated that SVM and ANN models were the most effective for tea plant classification. Chen et al. (2022) compared the performance of multilayer perceptron (MLP), SVM, random forest (RF), and PLSR using hyperspectral data from tea plants, developing a Tea-DTC model for evaluating drought resistance traits in 10 tea plant germplasm resources.

However, traditional machine learning methods are heavily reliant on manually selected features under controlled conditions, and their robustness tends to be limited, particularly in complex field environments. These methods often struggle to handle the challenges posed by the dynamic and variable real-world agricultural environments (Wang et al., 2021; Xia et al., 2023). Deep learning (DL) methods, however, excel in discovering patterns and hidden information from large datasets using neural networks (Liu et al., 2024). Unlike traditional machine learning, DL approaches are better suited for complex scenarios and require large amounts of data for training. Recent deep learning algorithms, such as Faster R-CNN, ResNet, and YOLO-based models, have demonstrated superior performance in crop yield estimation (Paudel et al., 2023; Li et al., 2024), growth monitoring (Wu et al., 2020; Li et al., 2024), and object detection for fruits and other crop targets (Sun et al., 2022; Wang et al., 2022; Rivera-Palacio et al., 2024). New deep learning modeling techniques, such as Transformer, have also begun to be applied to the development of agricultural deep learning models, for example, in rice disease identification (Lu et al., 2025b). In addition, Long Short-Term Memory networks with multi-head self-attention mechanisms have been employed for rice yield prediction (Lu et al., 2025a). Additionally, the integration of machine learning, deep learning, and plant phenotyping platforms, along with UAV technology, has resulted in the development of many new and efficient techniques. For instance, RGB and multispectral images were utilized to identify the tasseling stage of maize (Guo et al., 2021). Drone time-series images and a Res2Net50 model were used to identify five growth stages of rice germplasm, achieving good prediction results for the heading and flowering stages by combining RGB and multispectral images and developing a PLSR model (Lyu et al., 2023). Similarly, drone time-series images and deep learning models were applied for the dynamic monitoring of maize ear area (Yu et al., 2022). These advances have significantly contributed to the rapid and efficient extraction of plant information, facilitating accurate plant phenotyping.

YOLOv5, developed by Glenn Jocher et al (Jocher et al., 2022), is an improved version of YOLOv3. It is characterized by a relatively small model size and fast processing speed, making it suitable for mobile deployment. In recent years, YOLO-based algorithms, particularly YOLOv5, have been widely applied to object detection in agriculture, demonstrating superior performance on agricultural datasets (Farjon and Edan, 2024).

Several automatic detection models for various flowers, such as apple flowers, grapevine flowers, strawberry flowers, and litchi flowers, have been developed (Liu et al., 2018; Lin et al., 2020; Sun et al., 2021; Bhattarai and Karkee, 2022; Xia et al., 2023; Lin et al., 2024), as well as tea shoot detection models (Zhang et al., 2023; Bai et al., 2024; Chen et al., 2024; Wang et al., 2024; Wu et al., 2024). For instance, Wang et al. (2018) used color thresholding followed by SVM classification to estimate mango inflorescence area, employing Faster R-CNN for panicle detection. Lin et al. (2024) proposed a framework for counting flowers in litchi panicles and quantifying male litchi flowers, employing YOLACT++ for panicle segmentation and a novel algorithm based on density map regression for accurate flower counting. YOLOX was utilized by Xia et al. (2023) for tree-level apple inflorescence detection, achieving the highest AP50 of 0.834 and AR50 of 0.933.

To date, however, no models have been specifically developed to detect tea plant flowers or observe tea flower phenotypes. To fill this gap, we proposed a method for tea flowering quantification, comprising the TflosYOLO model and Tea Flowering Stage Classification (TFSC) model.

## 2 Materials and methods

### 2.1 Experimental design

**Figure 1:**
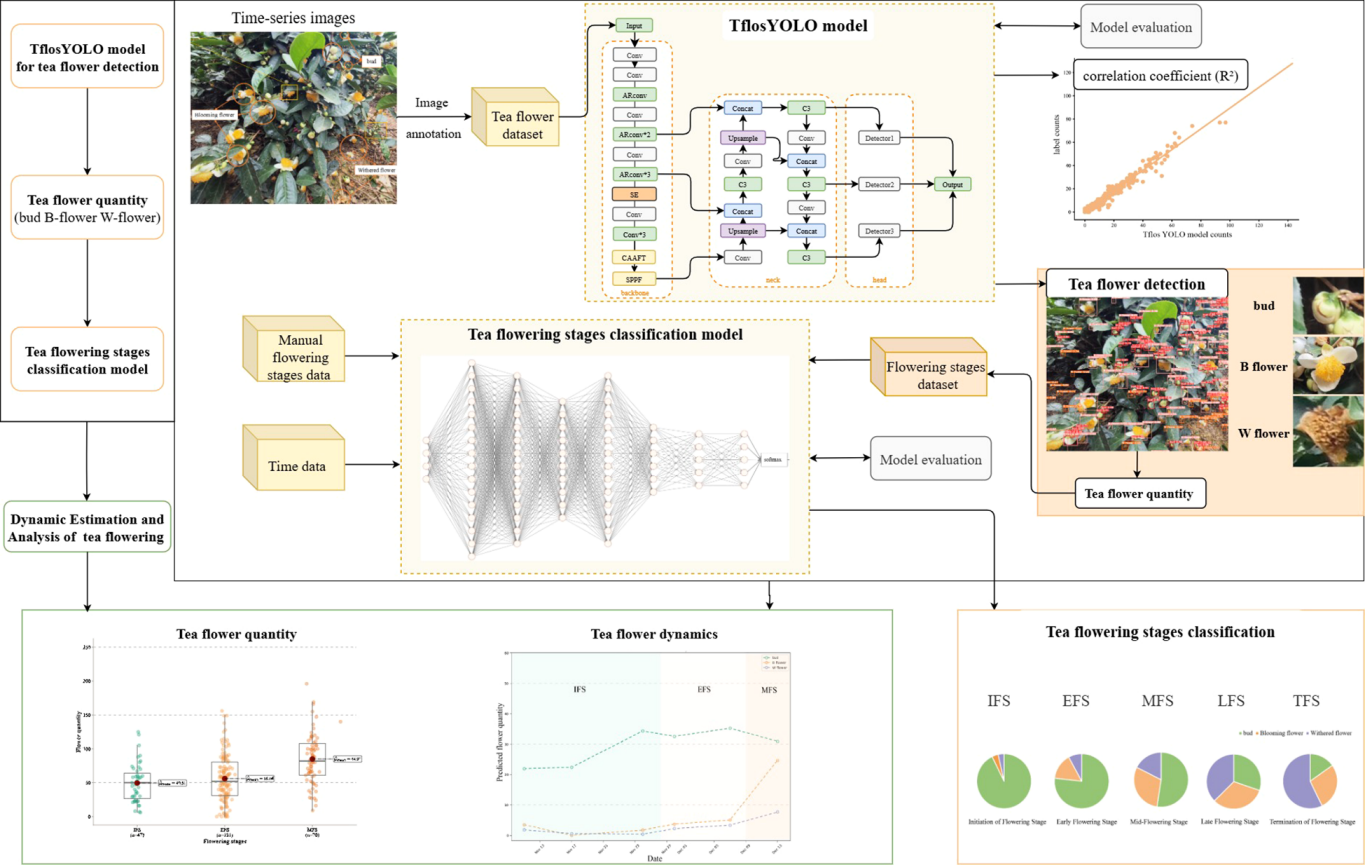
Overall framework for dynamic estimation and analysis of tea flowering.

The estimation of flower count and flowering period was achieved using time-series images of tea flowers by applying the TflosYOLO and TFSC model. The framework is shown in **Figure 1**. The process was outlined as fronts: mobile phone images of tea plant flowers were captured to establish a tea flower dataset, which was then used to train the TflosYOLO model. The TflosYOLO model provided the detection results for tea flower buds (bud), blooming flowers (B flower), and withered flowers (W flower), which were then used to output flower counts. The Tea Flowering Stage Classification model was used to determine the flowering stage [initial flowering stage (IFS), early peak flowering stage (EFS), mid peak flowering stage (MFS), late peak flowering stage (LFS), and terminal flowering stage (TFS).

### 2.2 Study site and materials

The experimental data used in this study were obtained in November–December 2023 and October–December 2024 at the National Tea Germplasm Research Garden (Hangzhou). Hangzhou is located in the southeastern region of China (29°–30°N and 118°–120°E), within a subtropical monsoon climate zone. Our research involved 29 tea accessions originating from different regions across the country; information on these accessions is provided in **Supplementary Tables S1** and **S2**.

### 2.3 Data acquisition

Tea plants in tea gardens are typically planted in rows with dense spacing between individual plants. Their flowers generally bloom predominantly on the sides of the plants. Considering this, a mobile phone was utilized for image capture, as mobile phone photography offers flexibility, making it feasible for large-scale, cost-effective, and precise phenotypic monitoring. The mobile phone captured images in RGB color format as JPG files. The image resolution was 3280 × 2464 pixels, with a 72-dpi setting. The actual area corresponding to the regions captured in each image was calculated using Fiji (Schindelin et al., 2012) and was approximately 3,690.33 cm^2^ (69.26cm × 53.28cm per image); the detailed method is shown in **Supplementary Figure S1**. In order to enhance the generalization ability of the model, images in the complex environments were collected in 2023–2024, including different lighting (e.g., backlight and frontlight), 29 tea accessions, various flowering densities, and both pruned and unpruned tea plants. In total, over 9,557 images were tokenized.

To evaluate the reliability of our approach as a substitute for traditional manual measurement and to explore the relationship between manual investigations and this framework, we conducted manual assessments of flower quantity and flowering stages after every image collection. The method of manual assessments is illustrated in **Supplementary Figure S2**.

### 2.4 Image annotation and dataset analysis

#### 2.4.1 Image annotation

The original images captured using mobile phones had a resolution of 3280 × 2464 pixels. The input image size for the YOLO model was determined based on the specific model configuration and task requirements. In this study, the input size for model training, validation, and testing was set to 640 × 640 pixels. To ensure compatibility with this input size and reduce computational cost, the original images were cropped into four sub-images, each with a size of 1640 × 1232 pixels. Image annotation was performed using LabelImg (Tzutalin, 2015) in YOLO format. The labeled images were divided into three datasets for training, validation, and testing, following a 6:2:2 ratio. Three categories were defined for annotation: buds, blooming flowers (B flower), and withered flowers (W flower) (**Supplementary Figure S3**). In total, 28,668 instances were labeled across 2,361 images in the tea flower dataset. Additionally, various additional test datasets were constructed after annotation to assess the model’s performance.

#### 2.4.2 Dataset for TflosYOLO model

Three datasets were constructed for the training, validation, and testing of the tea flower detection model. The final annotated dataset included 2,361 images with a total of 28,668 instances: 57% were buds, 25% were B flower, and 18% were W flower. Buds accounted for the majority of the instances, while withered flowers represented only 18%, indicating a class imbalance in the tea flower dataset (**Table 1**, **Supplementary Figure S4**). To ensure the reliability, generalization capability, and robustness of the tea flower detection model, the tea flower dataset included images from 26 tea accessions (**Supplementary Table S1**).

**Table 1:** The number of different classes in tea flower dataset.

Class	Training set (1,432 images)	Validation set (469 images)	Test set (460 images)	All instances
Bud	9,447	3,300	3,645	16,392
Blooming flower	4,303	1,410	1,538	7,251
Withered flower	2,905	996	1,124	5,025
All classes	16,655	5,706	6,307	28,668

Moreover, 31 additional test datasets were constructed to evaluate the model on various tea accessions, flowering stages, lighting conditions (backlight and frontlight), and unpruned tea plant images. Except for the unpruned test set, all test datasets were constructed using pruned tea plant images. The representative images and the number of images for 31 additional test datasets are provided in **Supplementary Figure S5**.

### 2.5 TflosYOLO model for tea flower detection

#### 2.5.1 TflosYOLO model and YOLOv5

Although YOLOv7 (Wang et al., 2023) and other YOLO models have also shown excellent performance on agricultural datasets, considering the trade-off between model accuracy and computational cost, we adopted YOLOv5m as the baseline model for further improvement, aiming to achieve accurate and efficient tea flower detection across various environments and accessions while minimizing computational costs. TflosYOLO is more suitable for flower detection and has an additional function for direct flower counting.

The YOLOv5 network consists of three main components: a) Backbone: CSPDarknet, b) Neck: PANet, and c) Head: YOLO Layer. Initially, data are passed through the CSPDarknet for feature extraction. Next, they are processed through PANet to achieve feature fusion. Finally, the YOLO layer performs object detection and classification, outputting the final results in terms of detected objects and their corresponding classes.

In the detection process of YOLO-based algorithms, the input image is processed to generate a feature map, which is divided into an S × S grid. For each grid cell, anchor boxes are scored, and boxes with low scores are discarded. Non-Maximum Suppression (NMS) is then applied to eliminate redundant boxes. Only the remaining boxes, along with their confidence scores, are retained and displayed. The confidence score is calculated as Equation 1:

(1) confidence score = Pr(object)∗IoU(pred, truth)∗Pr(class)

where

- Pr(object) represents the probability that an object exists,
- IoU represents the Intersection over Union between the predicted and ground truth boxes, and
- Pr(class) represents the probability that the predicted box belongs to each class.

IoU is the Area of Intersection, calculated as Equation 2:

(2) $$IOU=\frac{area(B_{P\;}\cap \;B_{gt})}{area(B_{P}\cup B_{gt})}$$

$B_{P\;}$ is the predicted bounding box, and $\;B_{gt}$ is the ground truth box.

The YOLOv5 loss function consists of three components: classification loss, objectness loss, and box loss. To compute the total loss, these three components are combined as a weighted sum, which is expressed as Equation 3:

(3) $$Loss\;=\;w_{box}l_{box}\;+w_{obj}l_{obj}+w_{cls}l_{cls}$$

-
$l_{box}$ is the box regression loss, which measures the difference between the predicted and ground truth box locations;
-
$l_{obj}$ is the object confidence loss, which evaluates the accuracy of the model’s object detection; and
-
$l_{cls}$ is the classification loss, which measures the model’s ability to classify the detected objects accurately.

#### 2.5.2 Challenges in tea flower detection

**Figure 2:**
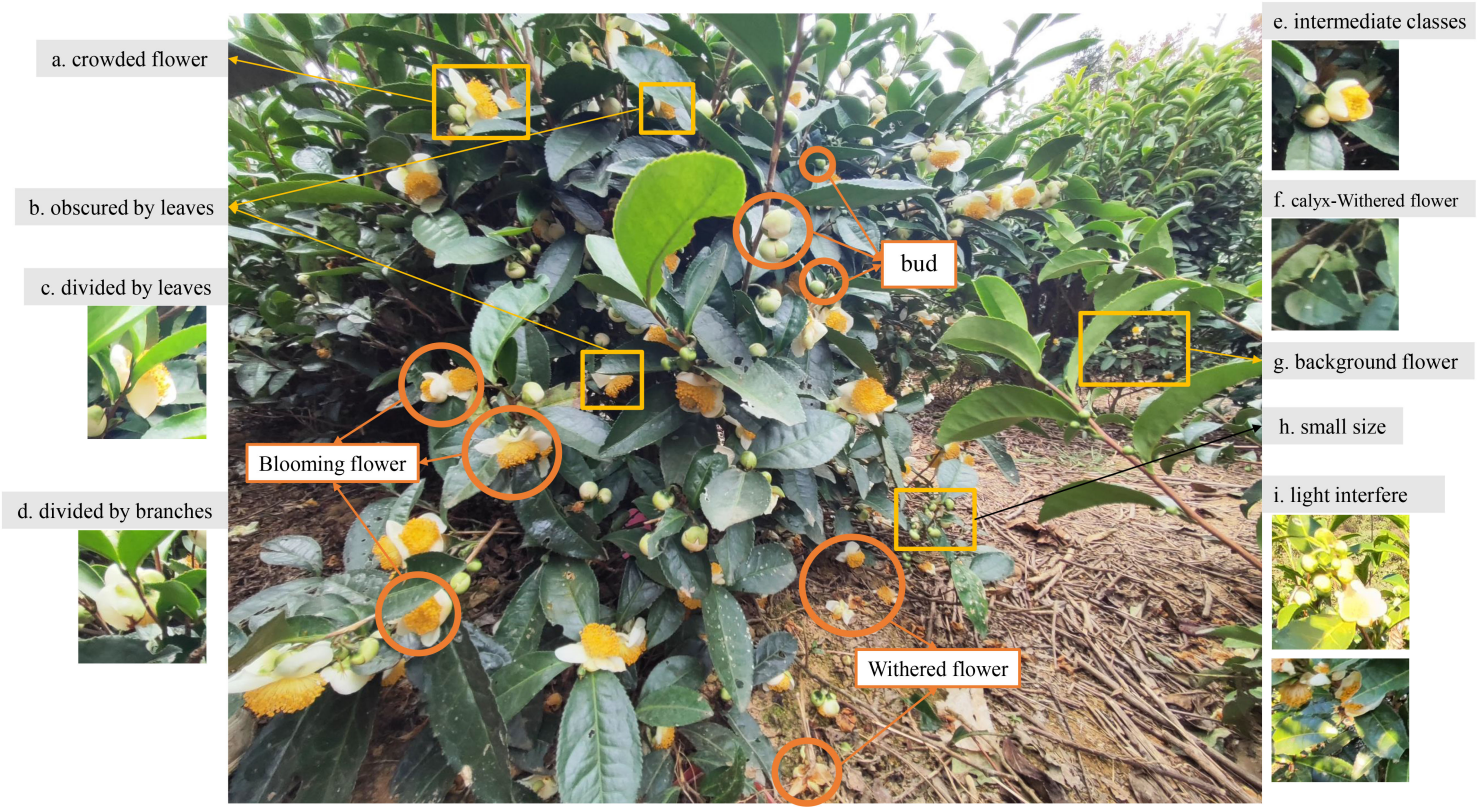
Examples of the inflorescences on tea plant and difficult issues in tea flower detection. **(a)** Crowded flowers obscured by each other. **(b)** Tea flower obscured by leaves. **(c)** Tea flower divided by leaves. **(d)** Tea flower divided by branches. **(e)** Intermediate classes. **(f)** Calyx belongs to withered flower, which can be easily detected as bud. **(g)** Background flowers that do not belong to the detected tree. **(h)** Small-sized detection target. **(i)** Light interference.

There are multiple challenges in tea flower detection, as shown in **Figure 2**. The field environment of a tea garden is complex, with varying light conditions, backgrounds, and other factors contributing to significant background noise. In addition to this, tea flowers are small and tend to grow on the side of the tea plant densely, with buds and flowers often obscuring each other, making them prone to being obstructed or fragmented by branches and leaves, and they are also easily influenced by background flowers. These factors make tea flower detection more challenging compared to the detection of fruits like apples (Xia et al., 2023). Furthermore, intermediate forms exist between buds, blooming flowers, and withered flowers, which are difficult to differentiate and can lead to a decrease in detection accuracy. Additionally, light interference, such as light spots, can cause buds to be misidentified. The imbalance among different flower categories is also one of the challenges, as the total number of tea buds and blooming flowers is significantly greater than the number of withered flowers. To address these challenges, this study proposed the TflosYOLO model, which aims to improve the accuracy of tea flower detection under various environmental conditions.

#### 2.5.3 Architecture of TflosYOLO model

**Figure 3:**
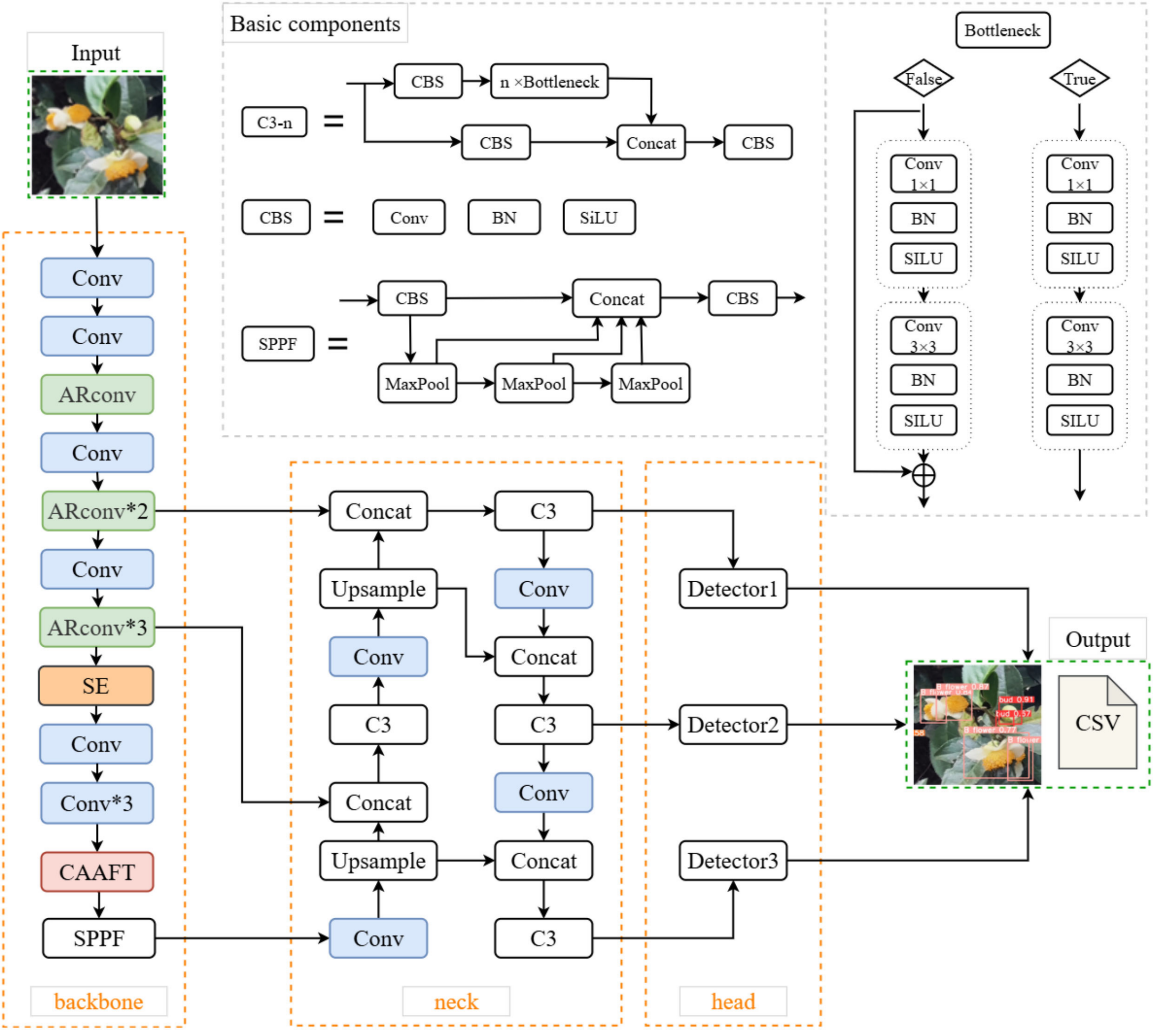
The model structure of TflosYOLO model. GP, global pooling; FC, fully connected layer.

The architecture of the TflosYOLO model (**Figure 3**) includes the backbone (CSPDarknet-53), the feature fusion neck, and the final detection layers. In this study, the YOLOv5m model is used as the baseline. The Squeeze-and-Excitation (SE) network attention module is integrated into the backbone of YOLOv5. Adaptive Rectangular Convolution (ARConv) is also used in the backbone of TflosYOLO. Additionally, Attention Free Transformer (CAAFT) is employed, which has lower computational complexity than traditional Transformer, making the model lighter and more efficient while improving performance. The additional function is added to output the flower counts directly as CSV. After improvement, the TflosYOLO model is more suitable for flower detection and flower counting.

The images are input into the TflosYOLO model, with the input size scaled to 640 × 640. The images pass through the main feature extraction network of the TflosYOLO model, generating various feature maps. These feature maps undergo further subsampling and feature fusion in the neck section, integrating shallow and deep features. The C3 modules at layers 18, 21, and 24 output feature maps of sizes 80 × 80, 40 × 40, and 20 × 20, respectively, for detecting small, medium, and large targets. Moreover, the TflosYOLO model outputs flower quantities of three types of tea flowers in CSV format for further analysis.

#### 2.5.4 Key improvements in the TflosYOLO model

This model introduces the integration of the SE Module, ARConv, CAAFT, and direct counting outputs. TflosYOLO can be regarded as a new version of YOLOv5 for better flower prediction and flower counting.

**Figure 4:**
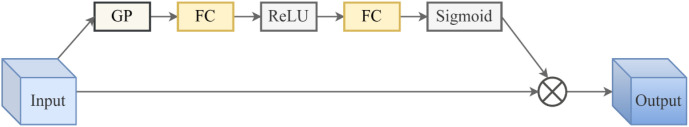
The structure of SE module. SE, squeeze-and-excitation.

The SE network module—a channel attention mechanism (Hu et al., 2018; Guo et al., 2022)—is added to the seventh layer of the YOLOv5 model. The structure of SE is shown in **Figure 4**. The SE module consists of two key steps: Squeeze and Excitation. It dynamically adjusts the weights of different channels by learning the relationships between channels in order to make the network focus on more important features while suppressing unimportant channels.

**Figure 5:**
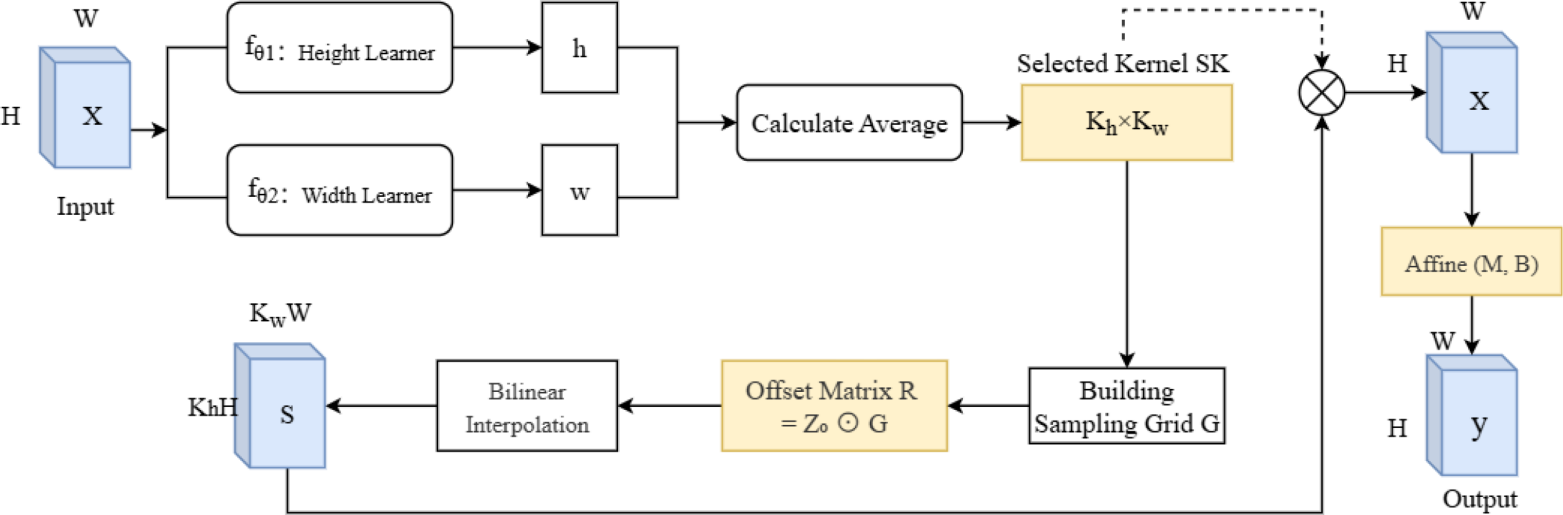
The structure of ARConv. ARConv, adaptive rectangular convolution.

ARConv: To enhance the model’s feature extraction capabilities and adapt to targets of different sizes, we applied Adaptive Rectangular Convolution in the backbone of the YOLO model. ARConv is a flexible and scalable convolutional module designed to enhance feature extraction for objects of varying sizes in images (Wang et al., 2025). Unlike standard or even deformable convolutions, ARConv adaptively learns both the height and width of the convolution kernel and dynamically determines the number and positions of sampling points. The structure of ARConv is illustrated in **Figure 5**. First, to learn the height and width of the convolution kernel, given an input feature map X, two subnetworks $f_{\theta_{1}}\;$ and $f_{\theta_{2}}\;$ predict the height and width feature maps (Equation 4):

(4) $$y_{i}\;=\;f_{\theta_{1}}\left(X\right),\;i\in \{1,2\}$$

These outputs are passed through a sigmoid activation and modulated as Equation 5:

(5) $$y_{i}\;=\;a_{i}\cdot Sigmoid\left(y_{i}\right)+b_{i}$$

where $a_{\text{i}}\;$ and $b_{i}$ are scale factors. The resulting maps h and w represent the predicted kernel height and width per pixel location, respectively.

After that, to derive the kernel dimensions $k_{\text{h}}$ and $k_{\text{w}}$, the average of the predicted *h* and *w* is computed and converted using Equation 6:

(6) $$k_{h}=\phi (\bar{h}),\;k_{w}=\phi (\bar{w})$$

where ∅ (x) = x− [x is even] ensures odd-valued kernels. Thus, the total number of sampling points is $k_{h}\cdot k_{w}$.

Next, generating the sampling map, a grid $G\in R^{k_{h}\cdot k_{w}}$ represents standard convolution offsets. For each center point p_0_, a scale matrix Z_0_ is generated from learned h_0_, w_0_ (Equation 7):

(7) $$Z_{0}\;=\;\left(\frac{h_{0}}{k_{h}},\;\frac{w_{0}}{k_{w}}\right)$$

The offset matrix $R\;\in \;R^{k_{h}\cdot k_{w}}$ is calculated via element-wise multiplication (Equation 8):

(8) $$R\;=\;Z_{o}⊙\;G$$

Sampling positions p_o_ + r_ij_ are typically non-integer, so bilinear interpolation is used to estimate the sampled feature values.

In the end, the interpolated features form the sampling map S. The final output is obtained by Equation 9

(9) y = SK ⊗ S ⊙ M ⊕ B

where SK is the selected convolution kernel, M and B are affine transformation matrices predicted via two lightweight subnets, ⊗ is convolution, ⊙ is element-wise multiplication, and ⊕ is element-wise addition.

**Figure 6:**
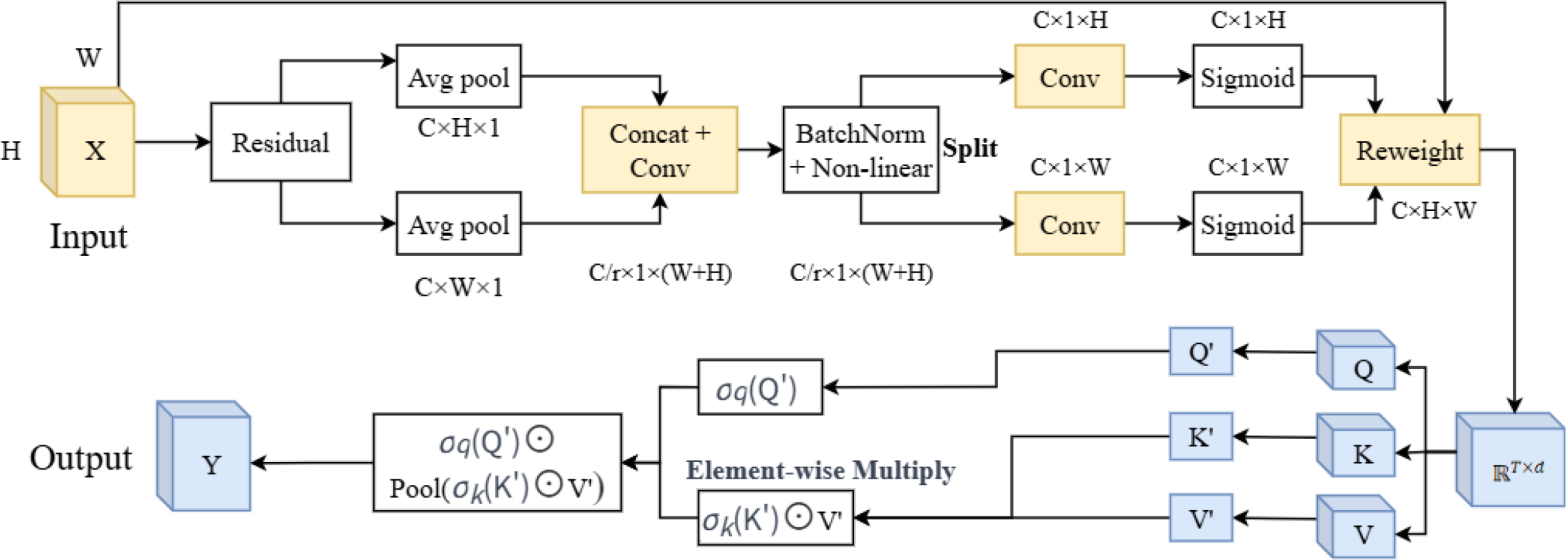
The structure of CAAFT. CAAFT, coordinate attention mechanism with the attention free transformer.

CAAFT: The CAAFT module integrates the Coordinate Attention mechanism with the Attention Free Transformer. Coordinate Attention (CA) factorizes global pooling into two 1D directions (horizontal and vertical), allowing the network to encode both channel interdependencies and precise spatial (positional) information with negligible computational overhead (Hou et al., 2021). Attention Free Transformer (AFT) entirely eliminates the dot-product attention mechanism, replacing it with a computationally lightweight structure based solely on element-wise operations and global (or local) pooling. It has lower computational complexity, making the model lighter and more efficient while improving performance (Zhai et al., 2021). The structure of the CAAFT module is shown in **Figure 6**.

Given an input tensor $X\;\in \;{\mathbb{R}}^{C\times H\times W}$, C is the number of channels, and H and W are respectively the height and width of the feature map. Instead of using standard 2D global pooling, CA performs 1D global pooling along spatial axes.

Horizontal pooling (along width), is calculated by Equation 10:

(10) $$z_{c}^{h}(h)\;=\;\frac{1}{W}\cdot \sum_{i=1}^{W}x_{c}\left(h,i\right),\;for\;h=1,\ldots ,H$$

Vertical pooling (along height), is calculated by Equation 11:

(11) $$z_{c}^{w}(w)\;=\;\frac{1}{H}\cdot \sum_{j=1}^{H}x_{c}\left(w,j\right),\;for\;w=1,\ldots ,W$$

This generates two direction-aware descriptors: $z^{h}\;\in \;{\mathbb{R}}^{C\times H\times 1}$ and $z^{w}\;\in \;{\mathbb{R}}^{C\times 1\times W}$. Then, the two descriptors are concatenated, the encoded feature is split along the spatial dimension, followed by sigmoid activation, and the input features are re-weighted.

After that, the matrix $R^{T\times d}$ applies linear transformations to obtain Q, K, and V (Equation 12).

(12) $$Q^{\prime }=QW_{Q},K^{\prime }=KW_{K},V^{\prime }=VW_{V}$$

Subsequently, element-wise operations and pooling are performed, and the output is computed as Equation 13:

(13) $$Y=\sigma_{q}(Q^{\prime })⊙Pool(\sigma_{k}(K^{\prime })⊙V^{\prime })$$

where

-
$\sigma_{q}$ and $\sigma_{K}$ are element-wise non-linearities,
- ⊙ denotes element-wise multiplication,
- pooling is performed along the sequence dimension, and
-
$\sigma_{q}(Q')$ serves as an output gate, controlling how each position’s query modulates the global context; $\sigma_{k}(K^{\prime })$ acts like a forget gate, determining how much each value contributes.

#### 2.5.5 Training details

The model was trained for 300 epochs with a batch size of 8 and a learning rate of 0.01, using the SGD optimizer. The input image was resized to 640 × 640 pixels. The experimental setup and environmental settings are detailed in **Table 2**. The training loss and verification loss of the TflosYOLO model are provided, as shown in **Supplementary Figure S6**.

**Table 2:** Experimental setup and environmental settings.

Operating system	Ubuntu 18.04
GPU	RTX 3080(10GB) *1
CPU	Intel^®^ Xeon^®^ Platinum 8255C
version	pytorch-cuda=11.8, Cuda 11.3, Python 3.8

#### 2.5.6 Model evaluation

In order to assess the model for tea flower detection, eight key performance indicators (KPIs) were adopted in this study. Precision and recall are commonly used evaluation metrics in deep learning Algorithm Evaluation, all of which are based on the confusion matrix (Ji et al., 2022). The confusion matrix is presented in **Supplementary Table S3**.

Precision is the proportion of true positives (TPs) in all detection-predicted positive samples (TP + FP). The formula is given by Equation 14:

(14) $$Precision\;=\;\frac{TP}{TP+FP}\;=\;\frac{TP}{all\;detections}$$

Recall is the proportion of TPs in all actual positive samples (TP + FN). The formula is given by Equation 15:

(15) $$Recall=\frac{TP}{TP+FN}=\frac{TP}{all\;actual\;positive}$$

F_1_-score combines precision and recall to measure the performance of a model. The formula is given by Equation 16:

(16) $$F1=2\times \frac{P\times R}{P+R}$$

where R is recall, P is precision, and C denotes class.

In object detection algorithms, Intersection over Union (IoU) is a commonly used metric to evaluate the accuracy of predicted bounding boxes against ground truth boxes. The formula is given by Equation 17:

(17) $$IOU=\frac{area(B_{P\;}\cap \;B_{gt})}{area(B_{P}\cup B_{gt})}\;$$

where B_p_ is the predicted bounding box, and $\;B_{gt}$ is ground truth box.

Average Precision (AP) is a key metric used to assess the performance of detection models over one class, reflecting the trade-off between precision and recall. Specifically, mean Average Precision (mAP) averages the AP across different classes; mAP0.5 refers to the mAP calculated at an IoU threshold of 0.5; mAP0.5–0.95 represents the mean Average Precision calculated across a range of IoU thresholds from 0.5 to 0.95. The formula is given by Equations 18, 19:

(18) $$AP=\int_{0>}^{1}P\left(R\right)dR$$

(19) $$mAP=\frac{\sum_{n=0}^{c}AP(C)}{C}$$

where R is recall, P is precision, and C denotes class.

Additionally, detection speed is used to evaluate detection time cost, while total parameters, Floating Point Operations Per Second (FLOPs), and model size are crucial for evaluating model complexity and computational cost.

In this study, we used the R^2^ coefficient to assess the strength of the correlation between the manually observed, annotated, and predicted tea flower numbers, further validating the reliability of the tea flower detection model. The formula for R^2^ calculation is provided in Equation 20:

(20) $$R^{2}=1-\frac{\sum_{i=1}^{n}{\left(y_{i}-{\hat{y}}_{i}\right)}^{2}}{\sum_{i=1}^{n}{\left(y_{i}-\bar{y}\right)}^{2}}$$

where n is the number of samples, $y_{i}$ is the manually observed or annotation flower quantity, and ${\hat{y}}_{í}$ is the predicted tea flower quantity from deep learning model, and $\bar{y}$ is the average of $y_{i}$.

### 2.6 Tea flowering stage classification model

#### 2.6.1 Flowering stage dataset construction

The tea plant flowering stage is categorized into five stages: IFS, EFS, MFS, LFS, and TFS. To construct the training and validation datasets, we utilized uncropped raw images of tea flowers collected in 2023. As the flowering periods of tea plants are influenced by climatic factors and can vary significantly between years, we incorporated tea flower images collected in 2024 to establish the test dataset. This test dataset, comprising 387 samples, aims to further validate the accuracy and generalizability of the flowering stage detection model.

Using the TflosYOLO model, the corresponding flower counts (including the number of flower buds, B flower, and W flower) for each image were estimated. Additionally, time data were incorporated. Manually recorded flowering stages were used as labels. Each image’s flower quantity, manually observed flowering stage, and time data constituted a flowering stage sample, collectively forming the original flowering stage dataset.

Subsequently, the original flowering stage dataset was preprocessed by first filtering out low-quality data. This involved removing images of varieties with insufficient flower counts, as they could not provide reliable flowering stage assessments. For the remaining samples from the same time and accession, the average value from every three samples was calculated to create a new sample. This approach mitigates the influence of extreme cases and reflects the overall flowering characteristics of the accession. Each sample was then manually labeled with tags that included IFS, EFS, MFS, LFS, and TFS. The 2023 flowering period data were divided into training and validation sets in an 8:2 ratio, while the 2024 images served as the test set.

#### 2.6.2 TFSC model design and training

The Flowering Stage Classification model is built using a seven-layer neural network and the flowering period dataset. ANN, also known as MLP, consists of fully connected layers. Each layer contains multiple artificial neural units (neurons). The model was implemented using PyTorch, with Rectified Linear Unit (ReLU) activation functions, softmax for classification, cross-entropy loss, and the Adam optimizer. The training parameters are shown in **Table 3**.

**Table 3:** Key training parameters.

Training samples	3,667
Validation samples	671
Test samples	387
Batch size	16
Learning rate	0.001
Epochs	80
Software version	pytorch-cuda=11.8, Cuda 11.3, Python 3.8

**Figure 7:**
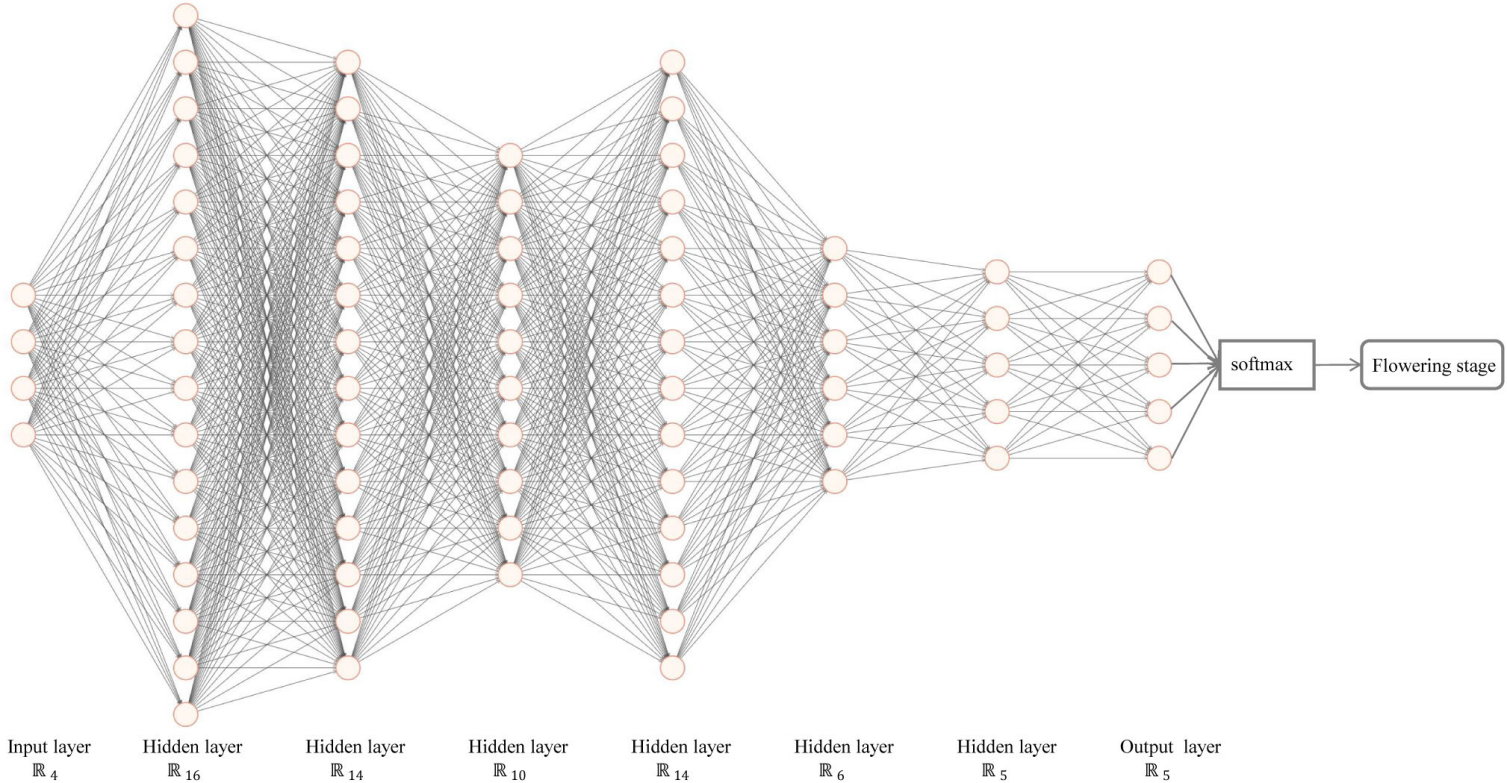
The tea flowering stage classification (TFSC) model.

The Flowering Stage Classification model is structured as a seven-layer neural network, shown in **Figure 7**. The input includes the number of buds, blooming flowers, and withered flowers, as well as time information. The labels are manually recorded flowering stage. After passing through six hidden layers and the softmax function for classification, the final output is the predicted probability of each flowering stage class.

The softmax function is calculated as Equation 21:

(21) $$\hat{y_{j}}=\frac{exp\left(o_{i}\right)}{\sum_{k}exp\left(o_{k}\right)}$$

where $\hat{y_{j}}$ represents the predicted probability, $o_{i}$ is the unnormalized prediction for the $i_{th}$ output, and *k* is the vector of predicted outputs. The softmax function ensures that the predicted outputs sum to 1, with each value in the range [0, 1].

The ReLU activation function is commonly used in artificial neural networks to introduce non-linearity and avoid issues such as gradient explosion and vanishing gradients. The ReLU function is defined as Equation 22:

(22) f(x) = max(0, x)

#### 2.6.3 Model evaluation

The accuracy is validated on the test set using the accuracy score function. The accuracy is calculated as Equation 23:

(23) $$ACC=\frac{TP+TN}{TP+TN+FP+FN}$$

where TP, TN, FP, and FN represent true positive, true negative, false positive, and false negative, respectively.

## 3 Results

### 3.1 TflosYOLO model performance and comparison

#### 3.1.1 TflosYOLO model performance for tea flower detection

The model performance was evaluated using the test dataset, and the results are summarized in **Table 4**. The TflosYOLO model can accurately detect and locate tea flowers. For the three categories, the mAP50 was 0.844, the precision was 0.788, the recall was 0.761, and the F_1_-score was 0.774. The mAP50 for flower buds, blooming flowers, and withered flowers all exceeded 0.78, with buds achieving the highest detection accuracy. The precision, recall, and F_1_-scores for bud and blooming flowers were all above 0.76. These results demonstrate that the model exhibits high accuracy and generalization capability. The model detection performance on one image is provided in **Supplementary Figure S7**, showing that TflosYOLO can accurately detect and locate tea flowers, even when they are obstructed by branches and leaves or when partial occlusions occur between flowers and buds.

**Table 4:** Performance of the TflosYOLO model based on test dataset.

Class	Precision	Recall	F_1_-score	mAP50	mAP50–95	Params/M	Model_size/M	GFLOPs
All classes	0.788	0.761	0.774	0.844	0.562	17.9	34.5	35.9
Bud	0.866	0.778	0.820	0.894	0.619			
B flower	0.782	0.76	0.771	0.851	0.551			
W flower	0.717	0.745	0.731	0.789	0.517			

#### 3.1.2 Evaluating the robustness of TflosYOLO model

**Figure 8:**
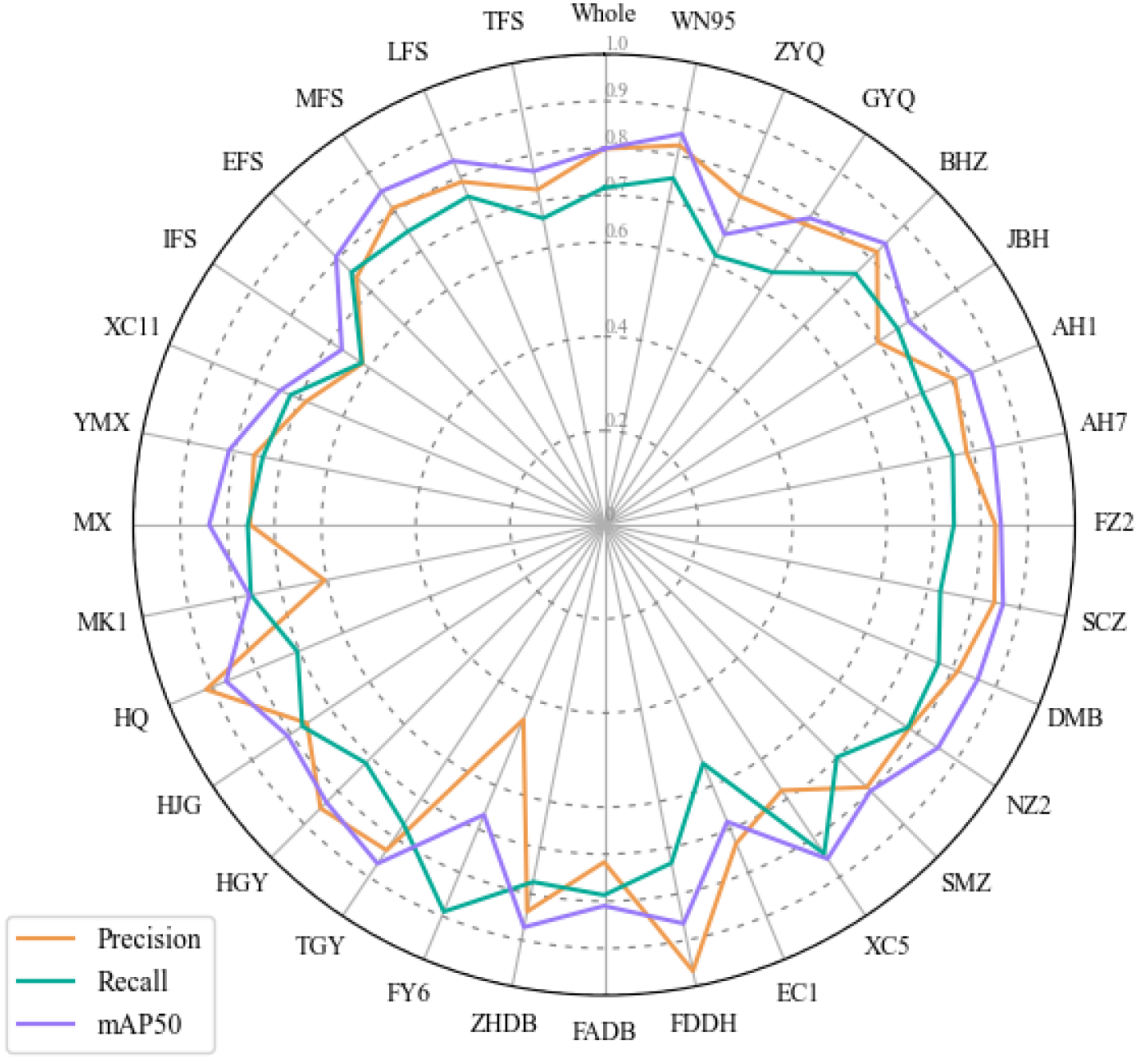
The performance of TflosYOLO model on 31 additional test sets.

To assess the robustness and generalization ability of the TflosYOLO model, 31 additional test datasets were used, covering 26 tea accessions and five flowering stage datasets: IFS, EFS, MFS, LFS, and TFS. The test results, as shown in **Figure 8**, present the precision, recall, and mAP50 values for the TflosYOLO model across 31 additional test datasets. For the many accessions, the mAP50 exceeded 0.75, and for several accessions, such as BHZ and AH1, it was above 0.8. However, the model performed slightly less effectively for some accessions, such as the mAP50 of EC1 and FY6 being less than 0.6, and the recall of EC1 being under 0.6. EC1 and FY6 plants both have very few flowers, which can be the main reason for the deviation. The model performed best during the Peak Flowering Stage (PFS) (including EFS, MFS, and LFS), while IFS and TFS had the lowest accuracy (**Figure 8**). In summary, the accuracy of the TflosYOLO model across most accessions, flowering stages under varying light conditions, remained above 0.7, indicating high robustness and generalization capability.

#### 3.1.3 Correlation analysis

**Figure 9:**
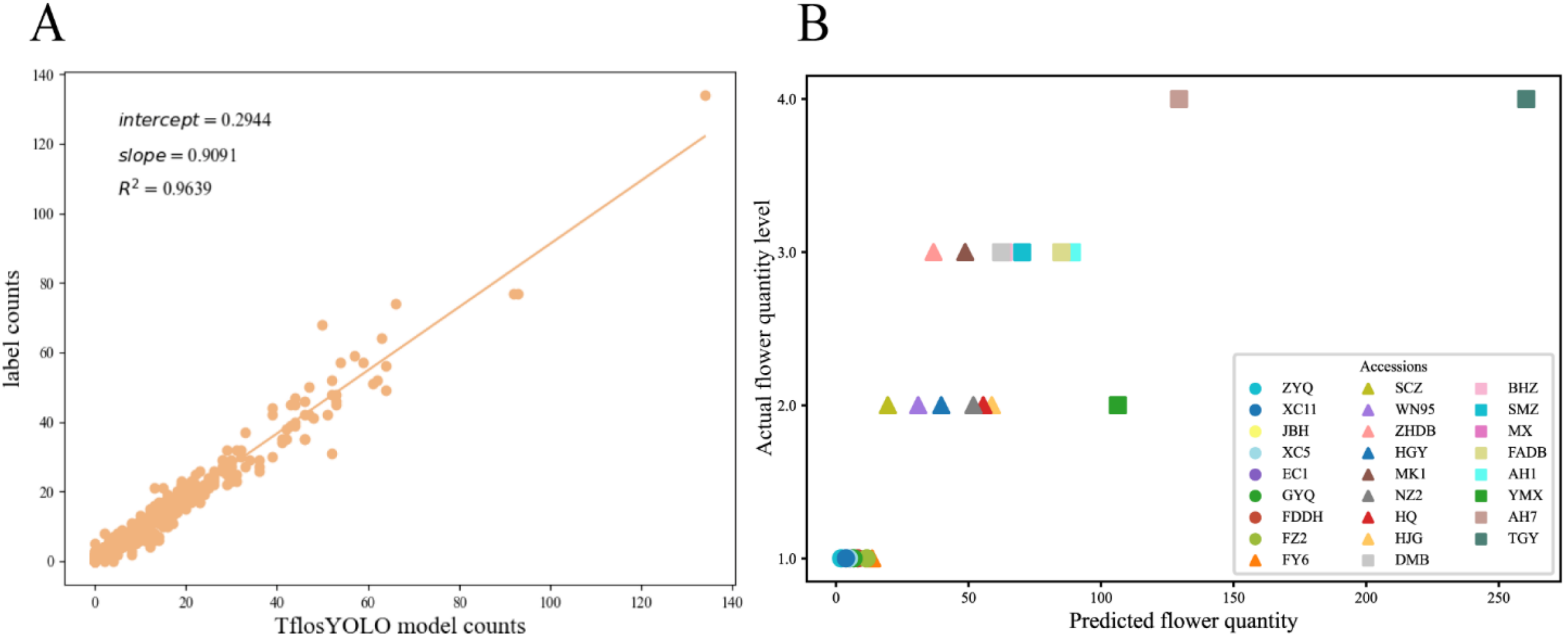
The correlation between the predicted flower count by TflosYOLO and the actual flower count. **(A)** The linear regression between the predicted flower count and the actual flower count (from labeled data). **(B)** The flower quantity comparison between the predicted flower quantity and actual flower quantity levels from traditional manual surveys.

To further evaluate the reliability of the TflosYOLO model, correlation analysis was conducted using the R^2^ coefficient. The correlation between the predicted flower count by TflosYOLO and the labeled flower count was computed based on the tea flower test dataset. The linear regression between the predicted flower count by TflosYOLO and the actual flower count (from labeled data) is shown in **Figure 9A**. The correlation coefficient (R^2^) for the predicted and actual flower count was 0.964, indicating a strong correlation between the predicted flower count and the actual count.

Additionally, the correlation between the predicted flower count and actual flower quantity levels from traditional manual surveys was analyzed. As shown in **Figure 9B**, the predicted flower count and flower quantity level from traditional manual investigation across 26 accessions were basically consistent.

#### 3.1.4 Ablation experiments of the TflosYOLO model

This study used YOLOv5m as the baseline model and incorporated various improvements into TflosYOLO to improve model performance in different environmental conditions. The ablation experiment was conducted based on the validation dataset. Compared to the YOLOv5m model, TflosYOLO demonstrated increased accuracy with lower computational costs (**Table 5**). The addition of the SE module, ARConv, and CAAFT further increased the recall, F_1_-score, mAP50, and mAP50–95, with no change in the number of parameters, model size, or Giga Floating-point Operations Per Second (GFLOPs).

**Table 5:** The evaluation result of the ablation experiment.

Model	Precision	Recall	F_1_-score	mAP50	mAP50–95	Params/M	Model_size/M	GFLOPs
YOLOv5m	0.759	0.685	0.720	0.760	0.499	20.9	40.2	47.9
YOLOv5+ARConv	0.786	0.704	0.743	0.791	0.485	13.9	28.1	32.7
YOLOv5+SE	0.797	0.701	0.746	0.795	0.478	6.7	13.0	15.5
YOLOv5+CAAFT	0.806	0.700	0.749	0.795	0.482	19.7	38.1	38.1
TflosYOLO	0.799	0.716	0.755	0.799	0.481	17.9	34.5	35.9

In general, these model enhancements were beneficial in addressing challenges under strong light and frontlight conditions and were effective in mitigating class imbalance issues. Furthermore, the Squeeze-and-Excitation networks and Attention Free Transformer contributed to model performance and resistance to background noise. Adaptive Rectangular Convolution enhances feature extraction for objects of varying sizes.

#### 3.1.5 Comparative performance of YOLO algorithms for tea flower detection

To compare the performance of the TflosYOLO model with other YOLO algorithms, we evaluated the Faster RCNN, YOLOv5 (n/s/m/l/x), YOLOv7 (yolov7-tiny/yolov7/yolov7x), and YOLOv8 (n/s/m/l/x) models based on a validation dataset. We trained the models using the same parameters, and the results are summarized in **Table 6**. Compared to Faster RCNN, YOLOv5, YOLOv7, and YOLOv8, TflosYOLO performed better in detecting tea flowers, achieving higher precision, recall, and mAP50–95 while requiring fewer computational resources. The table presents the average detection performance for the three classes: buds, blooming flowers, and withered flowers.

**Table 6:** Comparison of Faster RCNN, YOLOv5, YOLOv7, and YOLOv8 model performance.

Model	Precision	Recall	F_1_-score	mAP50	mAP50–95	Params/M	Model_size/M	GFLOPs
Faster RCNN	0.521	0.687	0.590	0.645	0.489	137.1	523.0	370.2
YOLOv5n	0.764	0.663	0.710	0.738	0.441	1.8	3.68	4.1
YOLOv5s	0.778	0.683	0.727	0.764	0.459	7.0	13.7	15.8
YOLOv5m	0.759	0.685	0.720	0.760	0.499	20.9	40.2	47.9
YOLOv5l	0.819	0.669	0.736	0.748	0.481	46.1	88.5	107.7
YOLOv5x	0.770	0.725	0.747	0.779	0.526	86.2	165	203.8
YOLOv8n	0.740	0.653	0.694	0.734	0.452	3.0	6.2	8.1
YOLOv8s	0.752	0.679	0.714	0.752	0.468	11.1	22.5	28.4
YOLOv8m	0.734	0.682	0.707	0.750	0.461	25.8	52.0	78.7
YOLOv8l	0.737	0.665	0.699	0.743	0.454	43.6	250	164.8
YOLOv8x	0.745	0.673	0.707	0.735	0.458	68.1	136.7	257.4
YOLOv7tiny	0.762	0.687	0.723	0.748	0.455	6.0	12.3	13.2
YOLOv7	0.761	0.720	0.740	0.778	0.490	37.2	74.8	105.1
YOLOv7x	0.726	0.701	0.713	0.744	0.452	70.8	142.1	188.9
TflosYOLO	0.799	0.716	0.755	0.799	0.481	17.9	34.5	35.9

### 3.2 Evaluation of the Tea Flowering Stage Classification model

**Figure 10:**
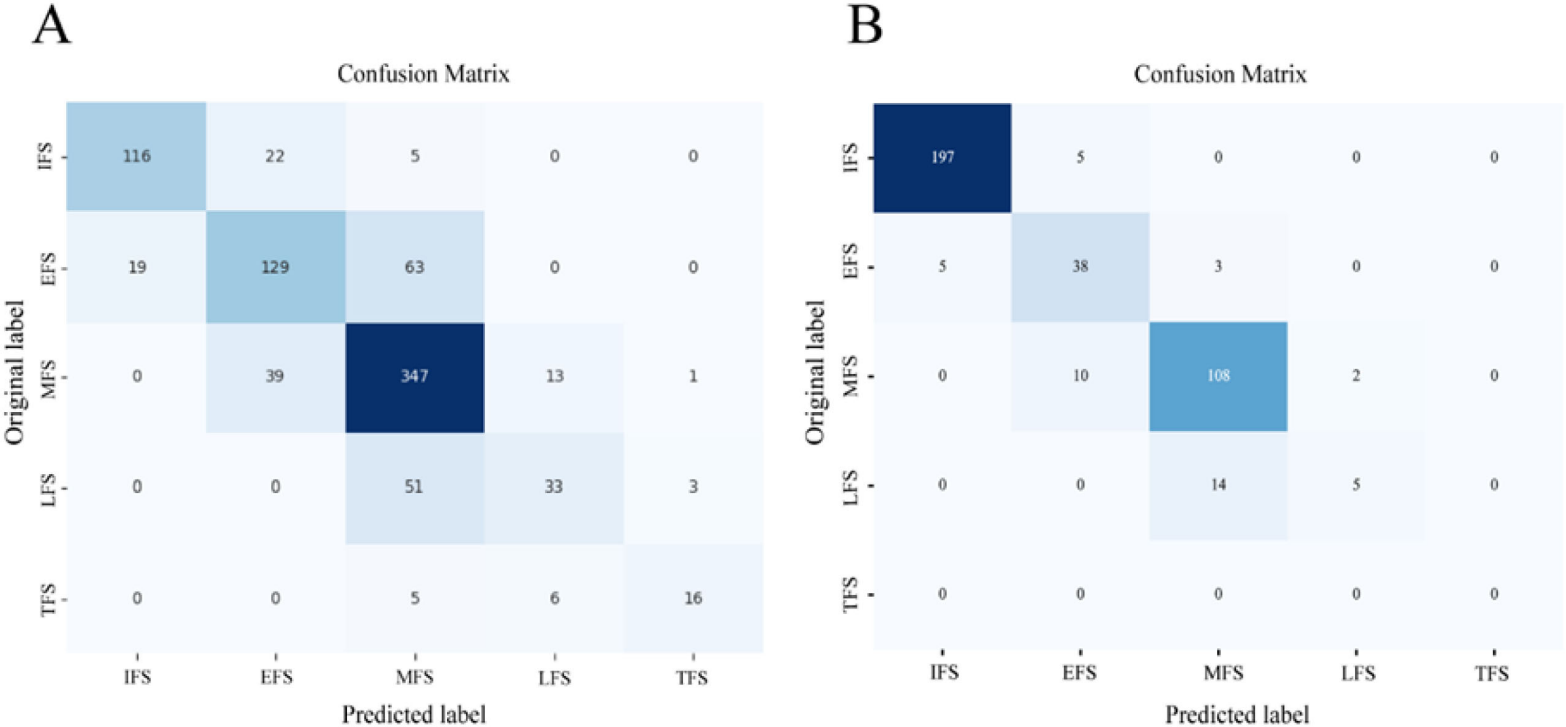
The confusion matrix of predicted flowering stages and manually recorded flowering stages. **(A)** The confusion matrix based on validation dataset. **(B)** The confusion matrix based on test dataset.

The TFSC based on ANNs achieved an accuracy of 0.738 and 0.899 on the validation dataset and test dataset, respectively. The confusion matrix (**Figure 10**) indicates that the classification of the flowering stages is prone to misclassification between adjacent stages. Specifically, there is frequent confusion between the EFS, MFS, and LFS, as the agricultural dataset contains a large number of intermediate periods and intermediate-type samples.

### 3.3 Application of the TflosYOLO+TFSC model in flower count and flowering period estimation

The TflosYOLO+TFSC model was used to perform dynamic flower counting and flowering period estimation. A time-series dataset constructed for observing tea flowering dynamics was used, including 29 tea accessions and five flowering stages in 2023–2024. The composition of this dataset is summarized in **Supplementary Table S4**. The tea flowering observation dataset contained a total of 5,029 and 4345 images in 2023 and 2024, respectively.

#### 3.3.1 Monitoring of tea flowering dynamics with flowering stage information

**Figure 11:**
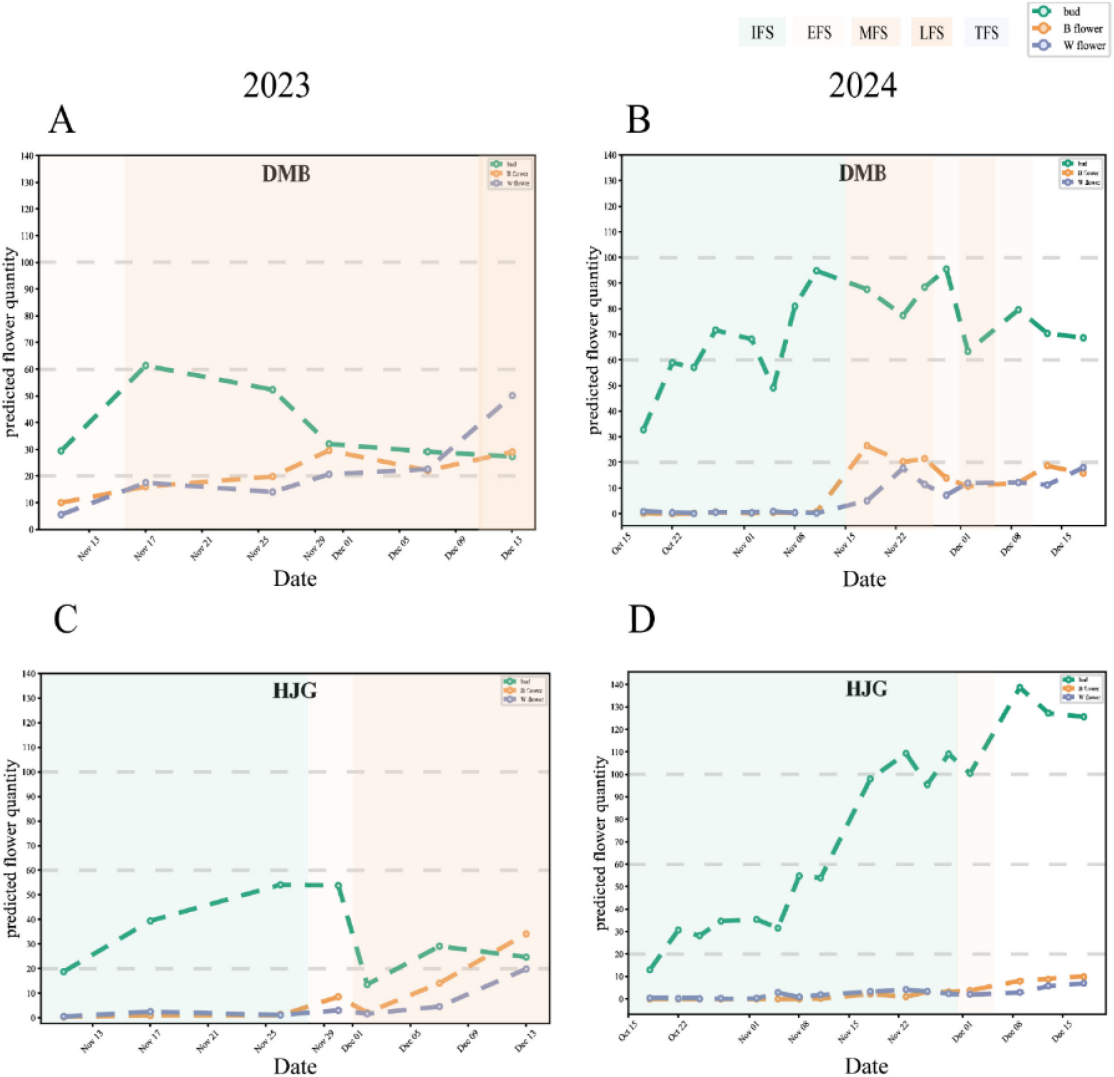
Tea flowering dynamics and flowering period information for two accessions in November–December 2023 and October–December 2024. **(A)** DMB in 2023. **(B)** DMB in 2024. **(C)** HJG in 2023. **(D)** HJG in 2024.

Using time-series images of 29 tea accessions in 2023, 2024, and the TflosYOLO + TFSC model, we monitored the flowering dynamics and tracked the changes in flowering stages. The reference for flowering dynamics visualization is shown in **Figure 11**. The tea flowering dynamics of other tea accessions in 2023 and 2024 are provided in **Supplementary Figure S8** and **Figures 9**, **10**. The flowering dynamics of different tea accessions exhibited distinct differences. In 2024, the flowering period of tea plants was generally later than that in 2023. Moreover, based on the results, the relatively early or late flowering of tea accessions is summarized in **Supplementary Table S6**. With the exception of BHZ, the flowering stages predicted by the model aligned with those recorded manually.

#### 3.3.2 Estimation of flower quantity across different tea accessions, years, and managements

**Figure 12:**
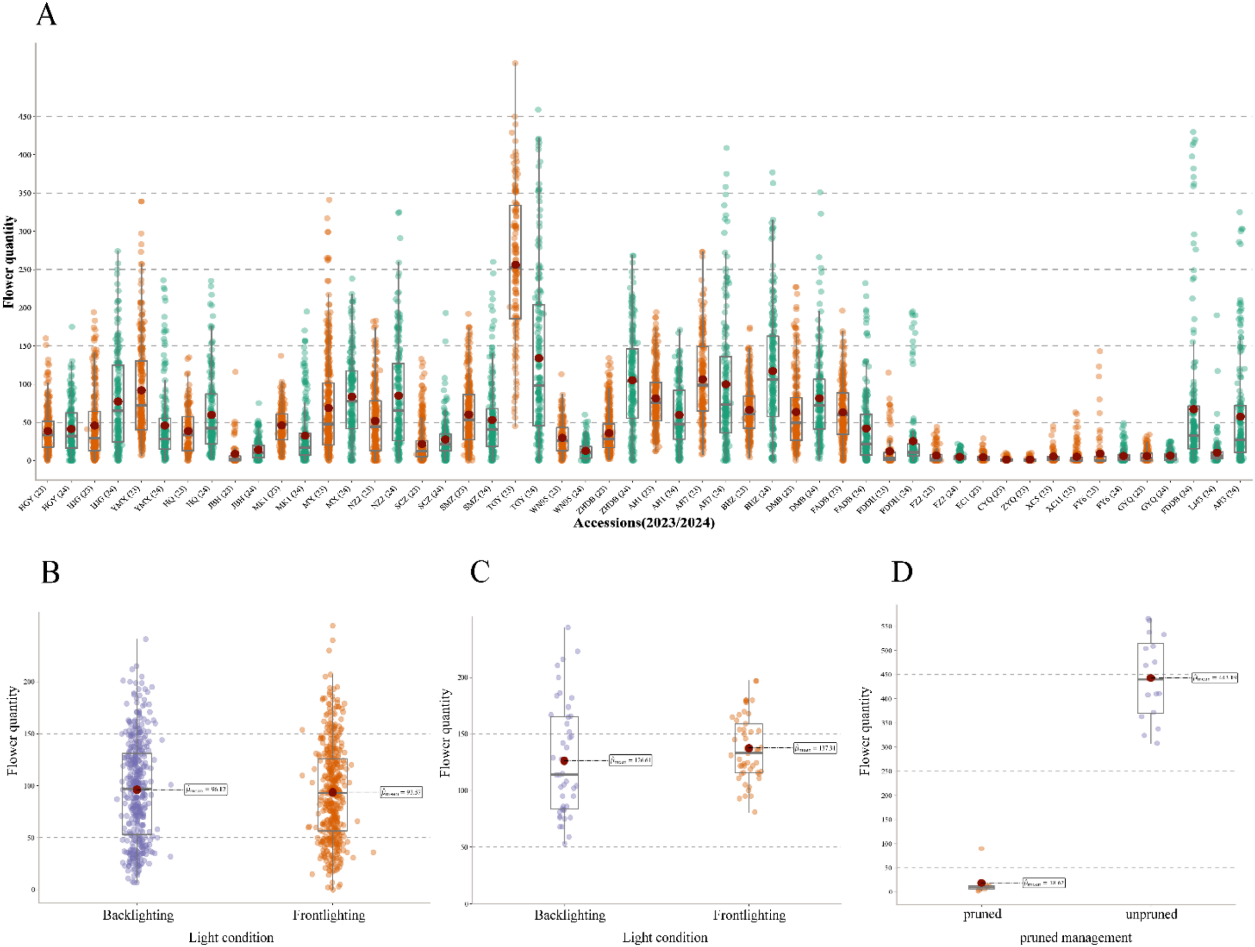
Estimation of flower quantity across different tea accessions, years, and managements. **(A)** Distribution of flower quantity across 29 accessions (2023 and 2024). **(B)** Flower quantity under frontlighting and backlighting conditions for tea plants from the same plot. **(C)** Flower quantity of Jin Xuan tea plants under frontlighting and backlighting conditions. **(D)** Distribution of flower quantity of pruned/unpruned management LJ43.

In this study, TflosYOLO was used to provide flower quantity data for each accession. The analysis and comparison of flower quantities across accessions were performed using data from the 2023–2024 PFSs (**Figure 12A**). Significant variability in flower quantity was observed across different tea accessions, and the flower quantity of the same accessions in 2023 and 2024 was relatively stable.

To further validate the robustness and reliability of the model, flower quantity under backlighting (BL) and frontlighting (FL) conditions was compared (**Figures 12B, C**). The flower quantities under backlighting and frontlighting for the same tea plants were similar, with no significant differences (p-value > 0.05). The results indicate that the TflosYOLO model demonstrated stable performance under both lighting conditions, unaffected by lighting variations. Additionally, a significant difference in flower quantity was observed between pruned and unpruned tea plants. The flower quantity of both pruned and unpruned LJ43 tea plants was compared, and unpruned LJ43 plants exhibited significantly higher flower quantities than the pruned ones, with a p-value < 0.01 (**Figure 12D**).

#### 3.3.3 Distribution of flower quantity across different tea flowering stages

**Figure 13:**
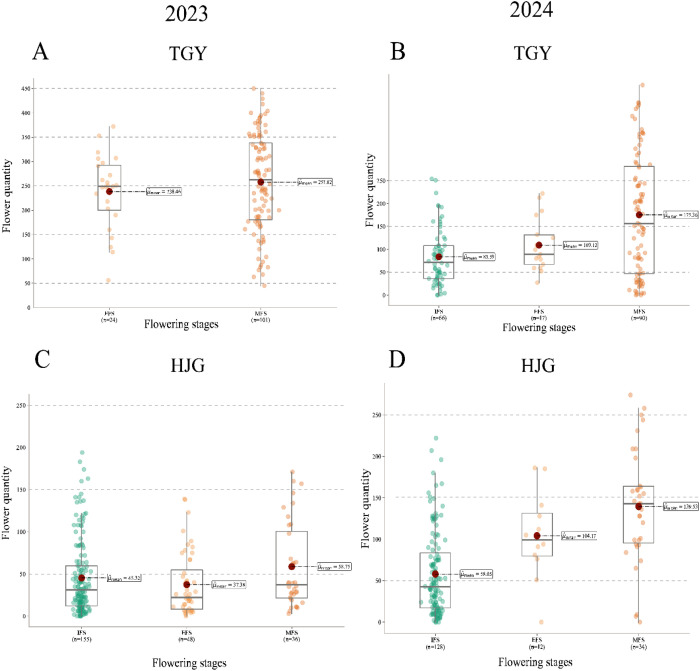
Flower quantity data for different flowering stage (IFS, EFS, MFS, LFS, TFS) across 2 accessions in 2023 and 2024. **(A)** TGY 2023; **(B)** TGY 2024; **(C)** HJG 2023; **(D)** HJG 2024. IFS, initial flowering stage; EFS, early peak flowering stage; MFS, mid peak flowering stage; LFS, late peak flowering stage; TFS, terminal flowering stage.

Furthermore, TflosYOLO was used to analyze the flower quantity for each flowering stage (IFS, EFS, MFS, LFS, and TFS) separately, the flower quantity of two selected accessions was analyzed and shown in **Figure 13**, and data of accessions from other provinces are provided in **Supplementary Figure S11**.

The flower quantity during different flowering stages varies significantly. For most tea accessions, they did not show significant differences in flower quantity between the three PFSs (EFS, MFS, and LFS), such as HJG (**Figure 13D**), but significant differences in flower quantity were observed among IFS, EFS, and MFS. In this case, conducting observations of flower quantity and flowering period over only a short period of time is likely to lead to errors.

## 4 Discussion

### 4.1 Importance of datasets

Agricultural datasets typically present challenges such as significant background noise and small object sizes, making the model performance very different from the evaluations conducted using datasets like COCO. For example, in this study, YOLOv5s outperformed the more computationally intensive YOLOv5l x and even YOLOv8. In the training and construction of deep learning models, such as YOLO, the representativeness and diversity of the dataset may be more crucial than improvements in the model architecture. The performance of the model can vary significantly across different accessions. Therefore, achieving good results on a single dataset does not guarantee consistent performance across all scenarios, and it is essential to test the model in different environments and with different accessions. Moreover, we validated the feasibility of employing the YOLOv5 computer vision model in complex field environments, demonstrating its applicability across different tea varieties. This validation allows us to assess the extent to which varietal differences influence model performance.

In this study, incorporating attention mechanisms such as SE, CBAM, and CEA led to significant improvements in cases with insufficient datasets, while their impact was less pronounced when the dataset was sufficiently large. Moreover, the composition of the dataset clearly affects the model performance. For instance, the predictions for the PFS (including EFS, MFS, and LFS) were the most accurate, particularly for the LFS, while performance during IFS and TFS was poorer. This is likely due to the training dataset predominantly consisting of images from the PFS.

### 4.2 Class imbalance

Regarding the issue of class imbalance in the dataset, several strategies were implemented to improve the overall performance of the model: (1) data augmentation, (2) incorporation of various attention mechanisms, and (3) expansion of the original dataset. First, multiple image augmentation techniques built into YOLOv5 were adopted, including image HSV-Hue augmentation, image HSV-Saturation augmentation, image HSV-Value augmentation, image rotation, and image mosaic. These augmentations noticeably enhanced model recognition performance compared with models trained without augmentation. Additionally, the integration of multiple attention mechanisms, such as the SE module used in this study, also improved accuracy for underrepresented classes.

Furthermore, increasing the dataset size is a highly effective strategy. When training with approximately 400 images, class imbalance resulted in significantly poorer detection performance for the minority class, withered flowers, compared with buds. However, after expanding the dataset to approximately 2,000 images, the performance gap between buds and withered flowers was substantially reduced.

### 4.3 Consideration of agronomic characteristics in quantifying different crop traits

When quantifying agronomic traits in crops, it is essential to account for specific agronomic characteristics. For example, tea flower quantity is greatly influenced by light exposure, and there are substantial variations in flower quantity across different tea plants of the same row. Thus, it is important to collect a sufficient number of images from various locations within the field. Additionally, tea accessions exhibit differences in morphology—ranging from small trees to shrubs—and the significant image disparities between pruned and naturally grown trees require models with high generalization and robustness.

### 4.4 Influence of plant size and weather on tea flower quantity

Flower quantity is strongly correlated with the size of the tea plant. To compare flower quantities across different accessions, it is important to ensure that the comparisons are made between plants of similar size and management practices. Additionally, tea flower quantity is influenced by weather conditions. Due to climatic differences between 2023 and 2024, the flowering dynamics of the same accession varied significantly, and the flowering period was generally later in 2024 than in 2023, as the extreme low temperatures in November and December 2023 were lower than those in November and December 2024. In the future, it would be valuable to combine tea flowering data with meteorological data to analyze the dynamics of tea plant flowering. Additionally, the observed flower quantity is significantly affected by both the flowering period and the timing of image acquisition. Consequently, observations made over a short time frame may not accurately reflect the true flowering dynamics.

### 4.5 Comparison with previous tea flower studies

Although previous tea flower studies conducted by manual survey involved fewer accessions, the overall flower quantity and flowering stage align with our findings. For instance, the flower quantity of accessions like MX and TGY was consistently high across different studies, and HJG displayed relatively high quantity.

### 4.6 Limitation

Previous studies have shown that the flowering period of tea plants can be influenced by regional climatic conditions. Therefore, relying solely on data from Hangzhou lacks verification across different regions and climatic environments, which limits the generalizability of the TFSC model. Continued investigation in this direction would be meaningful, as it could help further evaluate the model’s robustness and generalizability under diverse regional and climatic conditions.

## 5 Conclusions

This study proposed an effective framework for quantifying tea flowering, comprising the TflosYOLO model and TFSC model. Compared to traditional manual surveys and observations, this framework is more efficient and accurate. The TflosYOLO model demonstrates the ability to accurately detect tea flowers under various conditions, including different tea accessions, flowering stage, pruning practices, and lighting conditions. Its high robustness and generalization capability render it the only model currently suitable for detecting and counting tea flowers, achieving state-of-the-art (SOTA) performance in this domain. Additionally, the TFSC model consistently demonstrates an accuracy exceeding 0.73 across different years, indicating its high generalizability. TflosYOLO, combined with the TFSC model, enables the accurate estimation of flower count and flowering period across different accessions.

Based on TflosYOLO combined with the TFSC model, we found that there are differences in the flowering dynamics of various tea accessions. Accessions that are genetically related tend to exhibit more similar flower quantities and blooming periods. The flowering quantity and flowering period of the same accession can vary between different years due to changes in climate and management practices.

## Data Availability

The datasets presented in this study can be found in online repositories. The names of the repository/repositories and accession number(s) can be found below: https://github.com/sufie-mi/tea-flower-model.
