# Supplementary material for: TflosYOLO+TFSC: an accurate and robust model for estimating flower count and flowering period
Source: Front Plant Sci. 2025 Nov 14;16:1690413. doi: 10.3389/fpls.2025.1690413 (PMC12661935; doi:10.3389/fpls.2025.1690413)
Supplement: Supplementary file 1 [file DataSheet1.docx]

Supplementary data

**Manual assessments**

The tea plant flower quantity was classified into six levels, ranging from 0 to 5, with 0 indicating no flowers, 1 indicating almost no flowers, 2 representing a low overall flower density (with generally no more than 6 flowers on a single branch), 3 indicating a moderate overall flower density (with generally more than 6 flowers on a single branch), 4 representing a high overall flower density (with generally more than 10 flowers on a single branch and multiple flower buds at the same flowering point), and 5 representing the flower density higher than 4, where flowers are present in almost all positions on the tea plant row. (Fig. S2A.). Observations of flowering conditions were performed across the entire tea row, and flower quantity levels were recorded for each tea accession.

The flowering period of tea plants was categorized into stages: Initiation of Flowering Stage (IFS), Peak Flowering Stage (PFS), and Termination of Flowering Stage (TFS). The PFS was further divided into Early Peak Flowering Stage (EFS), Mid Peak Flowering Stage (MFS), and Late Peak Flowering Stage (LFS). Tea flowers were classified into three categories: buds, blooming flowers (B flower), and withered flowers (W flower). The determination of the tea flowering stage was based on the quantity and proportion of each flower type during different periods. For example, in this study the Initiation of Flowering Stage is defined as when over 80% of the flowers are buds, the Maximum Flowering Stage is characterized by more than 20% buds, over 40% blooming flowers, and less than 40% withered flowers, while the Termination of Flowering Stage is indicated by fewer than 10% buds and more than 40% withered flowers. A schematic diagram of the flowering stage classification is provided below. A schematic diagram of the flowering stage classification is provided in Fig. S2B.

**The actual area of images calculated using Fiji**

We collect several images with a scale every time we capture images, the side length of the black square is 1 cm, as shown in Fig. S1. The black square was used as scale and then calculate the actual area corresponding to the regions in the image by Fiji. After calculating the actual area of ten images，obtain the average value -3690.33 cm^2^, 69.26cm × 53.28cm.


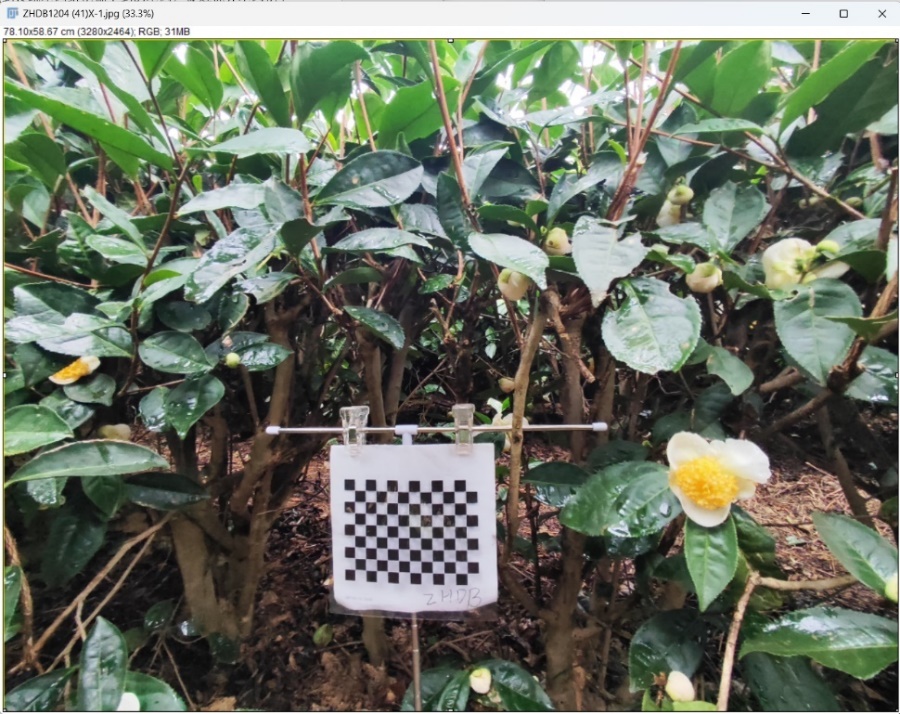


Fig. S1 Calculate the actual area based on the scale by Fiji.


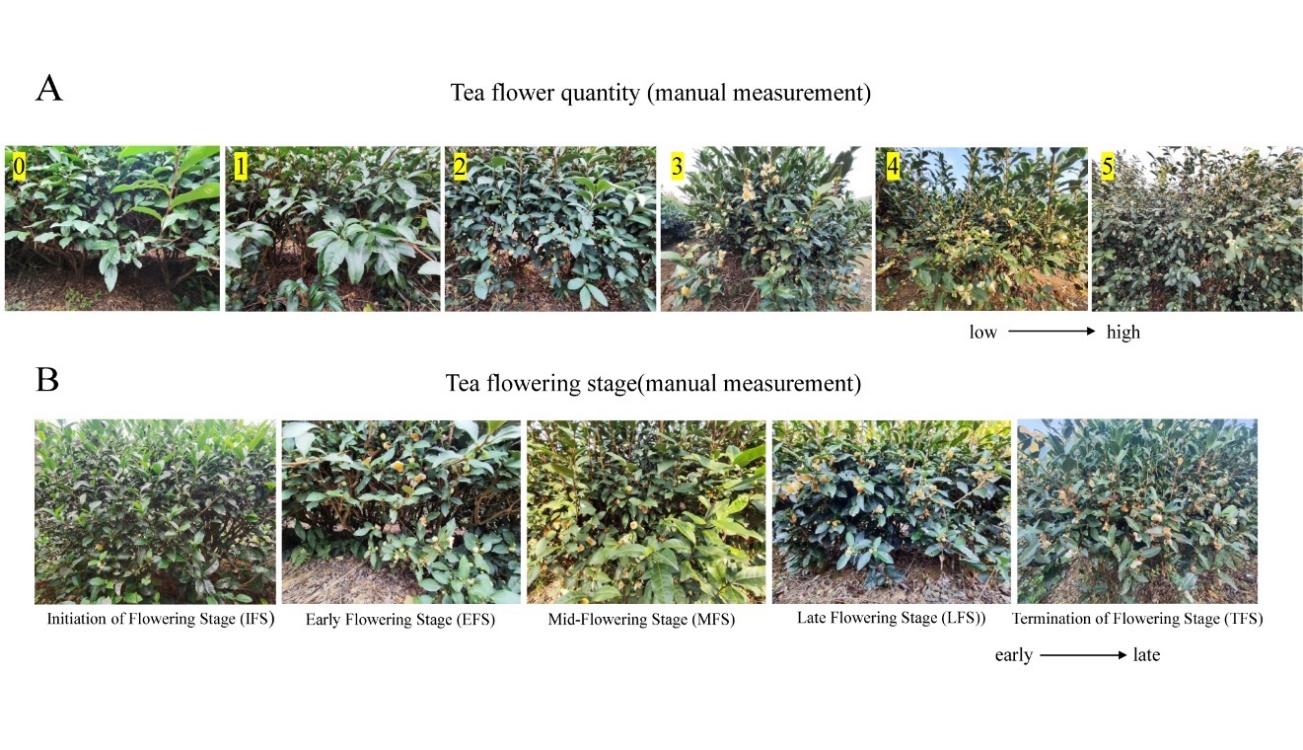
Fig. S2. Manual assessments of flower quantity and flowering stages. (A) tea flower quantity. (B) tea flower stage.


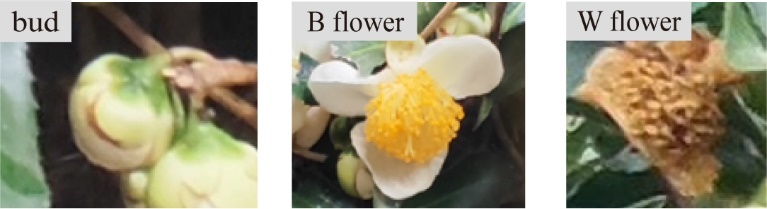


Fig. S3. Three categories of tea flower.


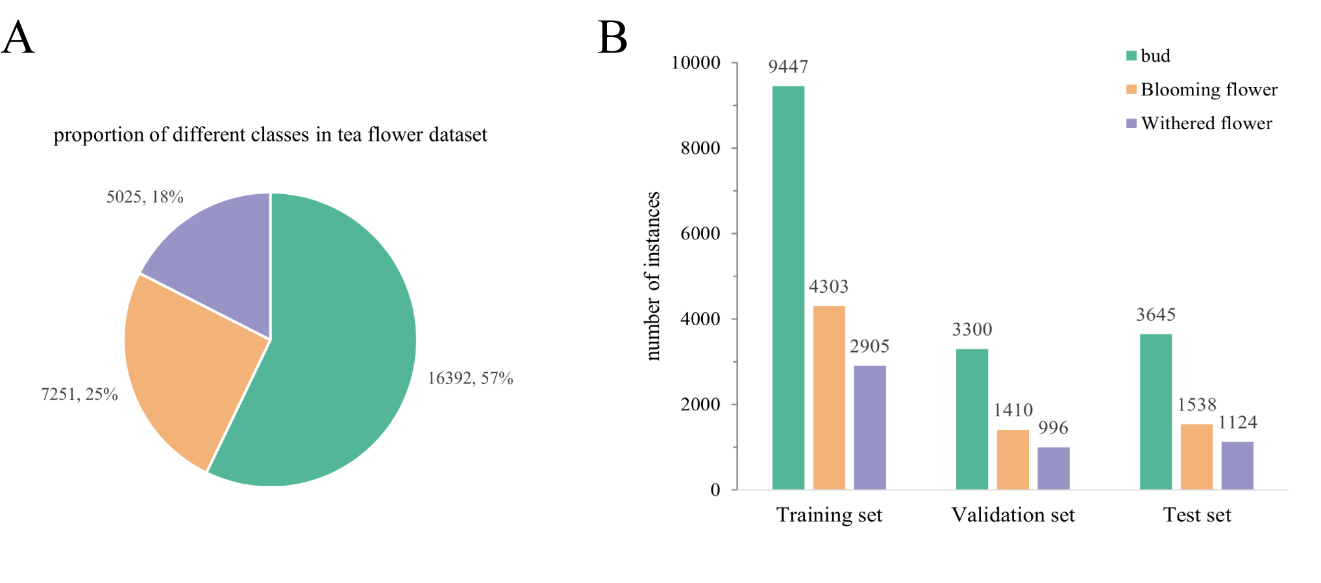


Fig. S4. (A) The proportion of buds, blooming flowers, and withered flowers within tea flower dataset (including training, validation, and testing dataset). (B) The quantity of buds, blooming flowers, and withered flowers within each dataset.


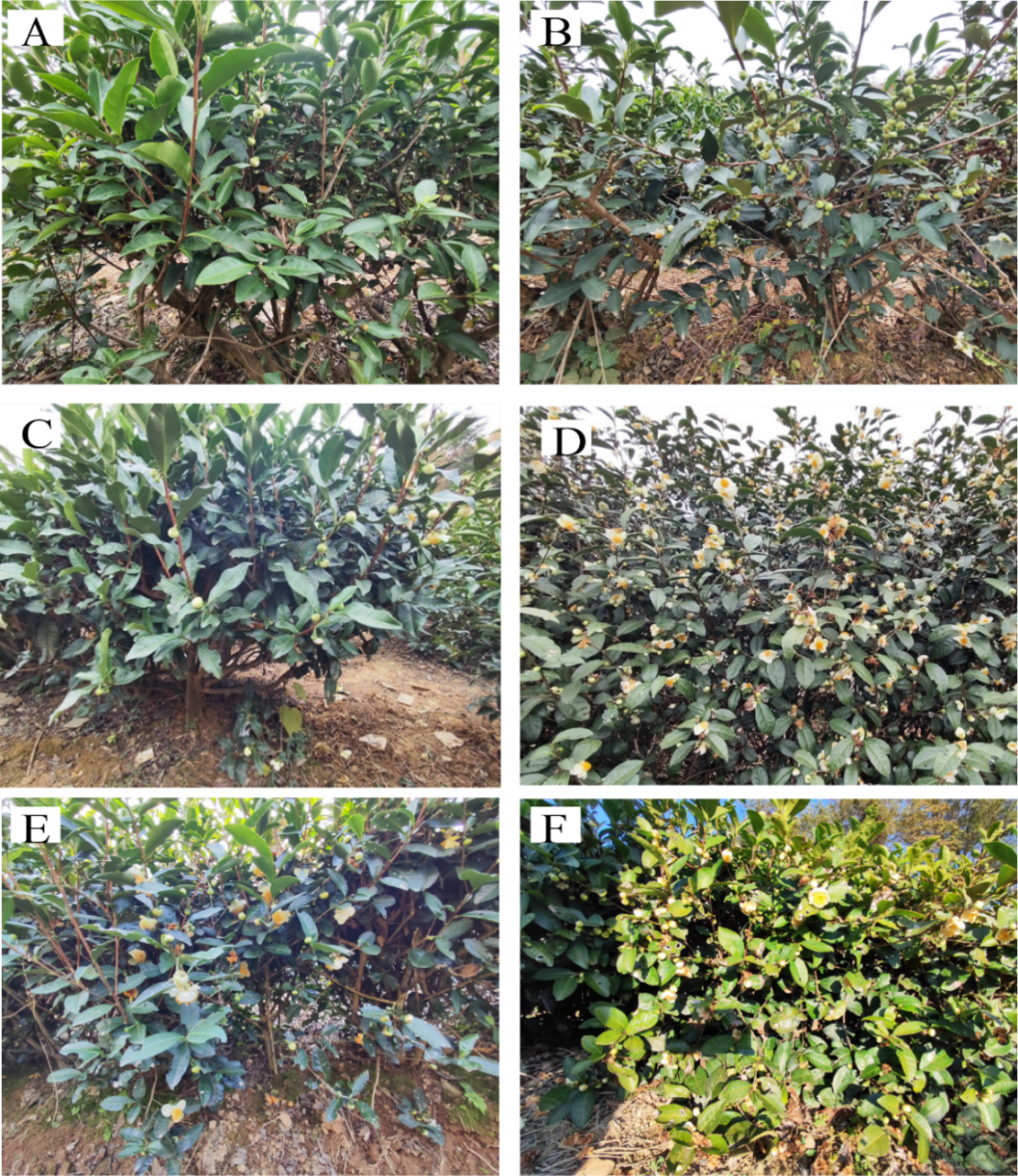


Fig. S5. (A) accession(BHZ). (B) accession(TGY). (C) pruned tea plants. (D) unpruned tea plants. (E) tea plants under backlight. (F) tea plants under bright frontlight.


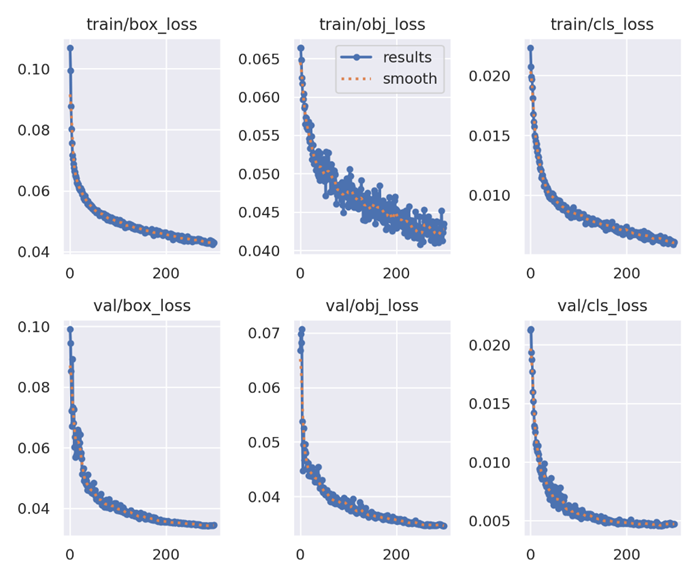


Fig. S6. Loss curve of TflosYOLO model during training.


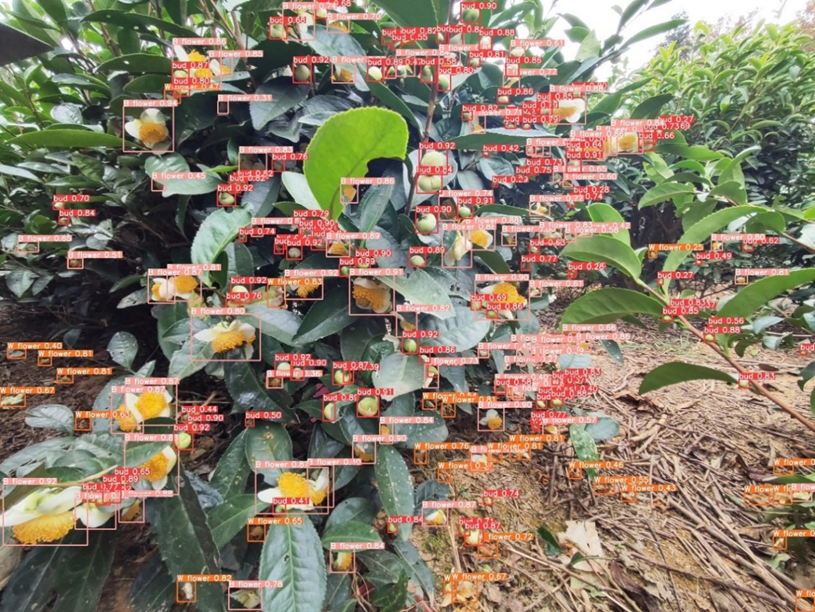


Fig. S7. TflosYOLO model detection performance on one image.


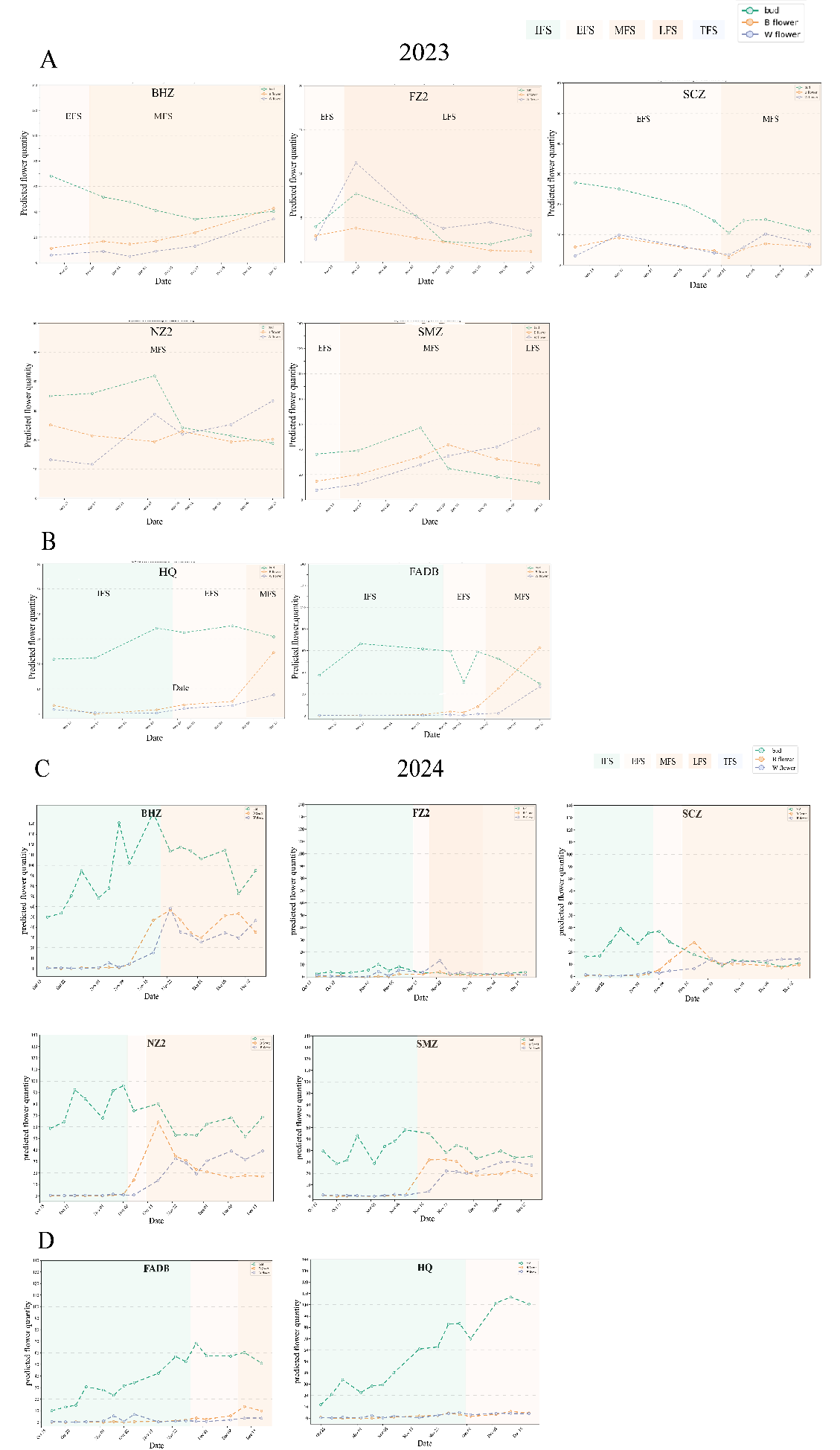


Fig. S8. The tea flowering dynamics and flowering stage information of 7 tea accessions in 2023 and 2024. (A) The tea flowering dynamics of tea accessions from Anhui and Huan province in 2023; (B) Fujian province in 2023; (C) Anhui and Huan province in 2024; (D) Fujian province in 2024.


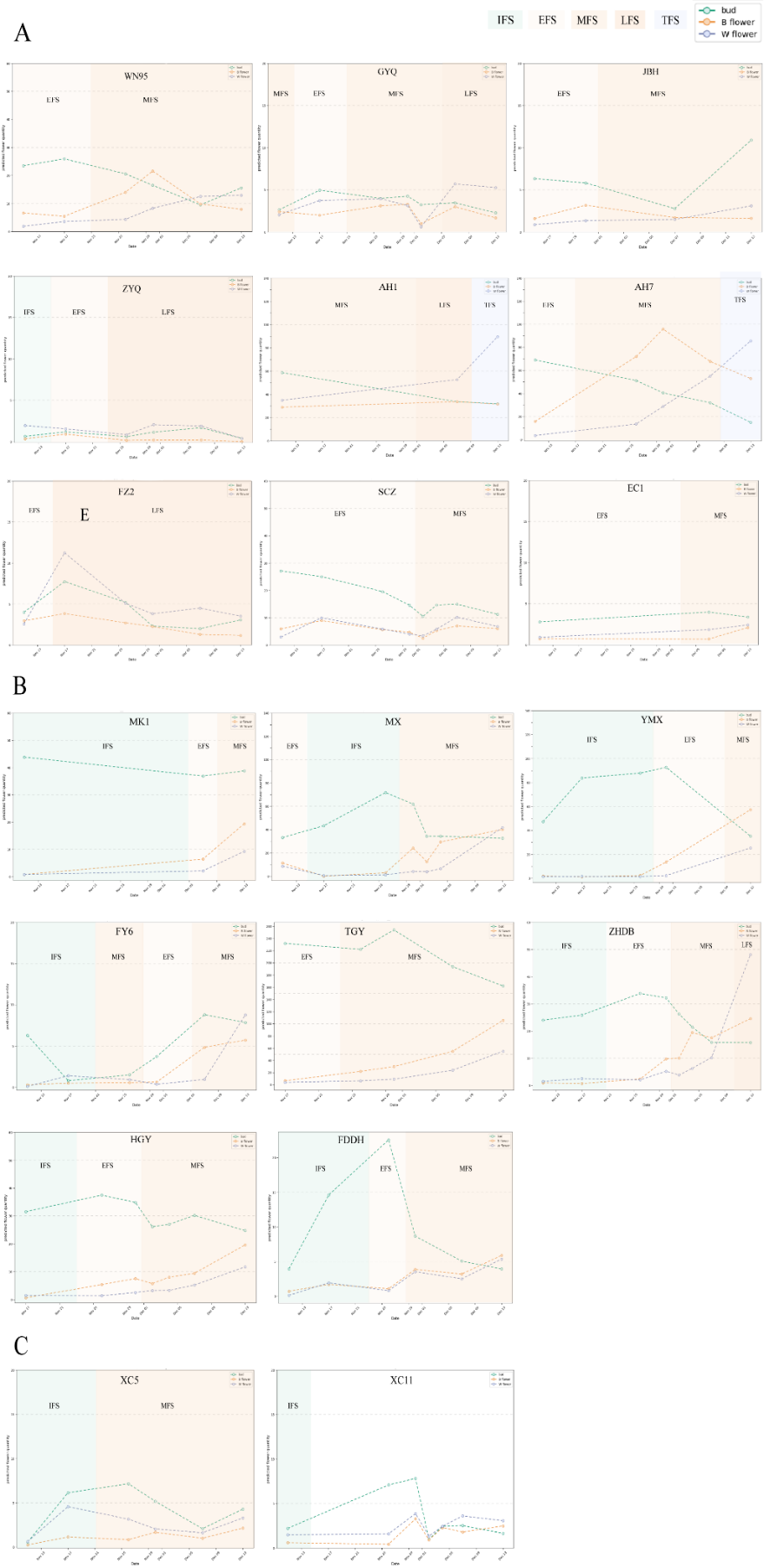


Fig. S9. The tea flowering dynamics and flowering stage information of 17 tea accessions in 2023. (A) The tea flowering dynamics of tea accessions from Anhui and Huan province (Southern Yangtze tea region); (B) The tea flowering dynamics of tea accessions from Fujian province (southern China tea region). (C) The accessions XC5 and XC11, based on their genetic lineage, were grouped in Jiangsu and Yunnan respectively.


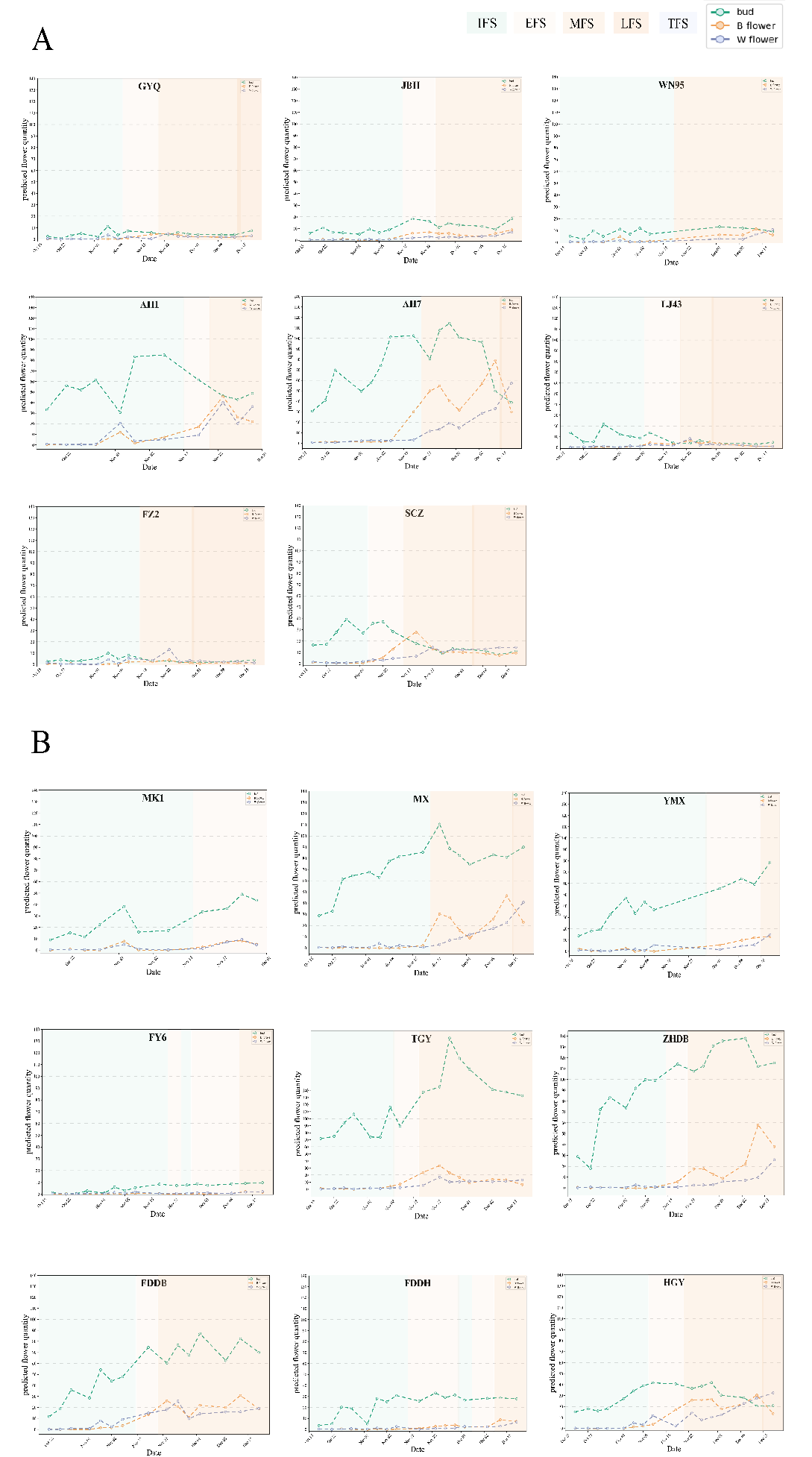


Fig. S10. The tea flowering dynamics of 17 tea accessions in 2024. (A) The tea flowering dynamics of tea accessions from Anhui, Zhejiang and Huan province; (B) The tea flowering dynamics of tea accessions from Fujian province.


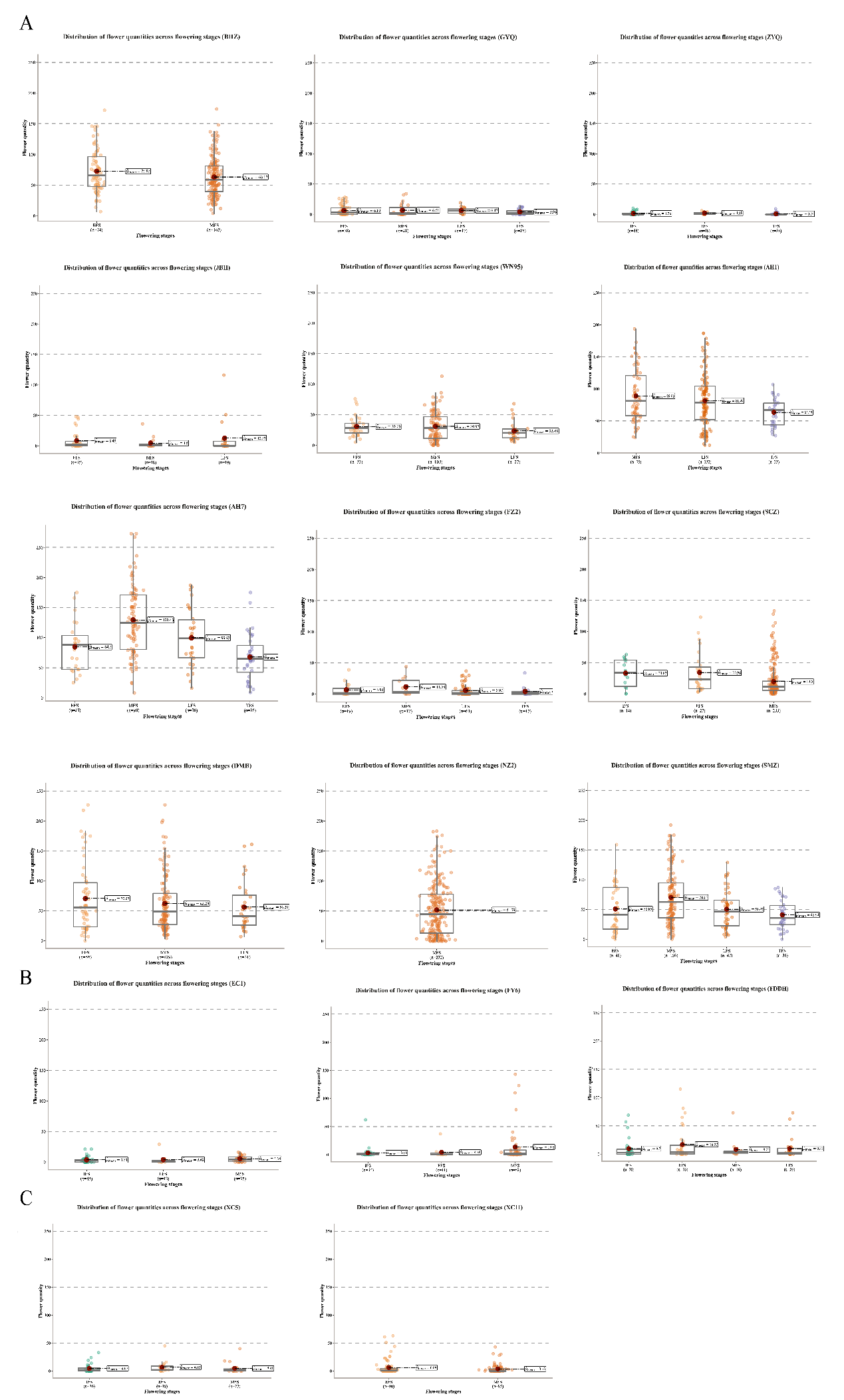


Fig. S11. Flower quantity data for each flowering stage (IFS, EFS, MFS, LFS, TFS) across accessions in 2023. (A) The tea flowering quantity of tea accessions from Anhui, Hunan and Jiangxi province (Southern Yangtze tea region); (B) The tea flowering quantity of tea accessions from Fujian province (southern China tea region). (C) The accessions XC5 and XC11, based on their genetic lineage, were grouped in Jiangsu and Yunnan, respectively.

Table S1. Data samples of different accessions in tea flower dataset.

| Origin province | Accession | Training set | Validation set | Test set | All |
| --- | --- | --- | --- | --- | --- |
| Hunan | Wannong 95 (WN95) | 32 | 17 | 20 | 69 |
|  | Zhuyeqi (ZYQ) | 50 | 20 | 5 | 75 |
|  | Gaoyaqi (GYQ) | 99 | 29 | 5 | 133 |
|  | Baihaozao (BHZ) | 30 | 18 | 33 | 81 |
|  | Jianbohuang (JBH) | 11 | 6 | 5 | 22 |
| Anhui | Anhui 1 (AH1) | 44 | 17 | 28 | 89 |
|  | Anhui7 (AH7) | 38 | 7 | 14 | 59 |
|  | Fuzao 2 (FZ2) | 29 | 3 | 12 | 44 |
|  | Shuchazao (SCZ) | 63 | 15 | 23 | 101 |
| Jiangxi | Damianbai (DMB) | 73 | 18 | 31 | 122 |
|  | Ningzhou 2 (NZ2) | 151 | 53 | 18 | 222 |
|  | Shangmeizhou (SMZ) | 156 | 55 | 36 | 247 |
| Jiangsu | Xicha 5 (XC5) | 65 | 11 | 15 | 91 |
| Fujian | Echa 1 (EC1) | 18 | 8 | 8 | 34 |
|  | Fuding Dahaocha (FDDH) | 29 | 6 | 8 | 43 |
|  | Fu’an Dabaicha (FADB) | 39 | 13 | 12 | 64 |
|  | Zhenghe Dabaicha (ZHDB) | 73 | 20 | 29 | 122 |
|  | Fuyun 6 (FY6) | 14 | 9 | 9 | 32 |
|  | Tie guanyin (TGY) | 36 | 4 | 10 | 50 |
|  | Huang guanyin (HGY) | 43 | 11 | 22 | 76 |
|  | Huang jingui (HJG) | 55 | 13 | 23 | 91 |
|  | Huangqi (HQ) | 33 | 8 | 12 | 53 |
|  | Mingke 1 (MK1) | 43 | 14 | 10 | 67 |
|  | Maoxie (MX) | 73 | 30 | 29 | 132 |
|  | Yuemingxiang (YMX) | 50 | 21 | 19 | 90 |
| Yunnan | Xicha 11 (XC11) | 76 | 35 | 12 | 123 |
| **all** |  | **1423** | **461** | **448** | **2332** |

Table S2. tea accessions information (not include in Table S1)

| accessions | abbreviation |
| --- | --- |
| Fuding Dabaicha | FDDB |
| Longjing 43 | LJ43 |
| Anhui 3 | AH3 |

Table S3. Confusion matrix.

|  |  | Actual class | |
| --- | --- | --- | --- |
|  |  | 1 | 0 |
| Predicted class | 1 | True Positive (TP) | False Positive (FP) |
|  | 0 | False Negative (FN) | True Negative (TN) |

Table S4. Data samples of different accessions and Flowering Stage in time-series dataset across 2023 and 2024. (Data samples are shown as: 2023/2024).

| Origin province | Accession |  | PFS | | |  | All stages |
| --- | --- | --- | --- | --- | --- | --- | --- |
|  |  | IFS | EFS | MFS | LFS | TFS |  |
| Hunan | WN95 | 0/92 | 33/31 | 103/42 | 27/0 | 0/0 | 163/165 |
|  | ZYQ | 44/0 | 19/0 | 0/0 | 0/0 | 35/0 | 98/0 |
|  | GYQ | 0/79 | 78/11 | 28/72 | 17/0 | 25/0 | 148/162 |
|  | BHZ | 0/93 | 74/0 | 163/90 | 0/0 | 0/0 | 237/183 |
|  | JBH | 0/86 | 35/45 | 18/44 | 19/0 | 0/0 | 72/175 |
| Anhui | AH1 | 0/60 | 0/84 | 74/33 | 132/0 | 33/0 | 239/177 |
|  | AH3 | 0/88 | 0/12 | 0/78 | 0/0 | 0/0 | 0/178 |
|  | AH7 | 0/77 | 23/0 | 90/81 | 30/12 | 36/0 | 179/170 |
|  | FZ2 | 0/78 | 19/12 | 12/34 | 61/45 | 17/0 | 109/169 |
|  | SCZ | 14/69 | 27/19 | 233/80 | 0/11 | 0/0 | 274/179 |
| Jiangxi | DMB | 0/87 | 56/24 | 129/70 | 31/0 | 0/0 | 216/181 |
|  | NZ2 | 0/78 | 0/11 | 232/87 | 0/0 | 0/0 | 232/176 |
|  | SMZ | 0/93 | 46/0 | 156/93 | 47/0 | 36/0 | 285/186 |
| Jiangsu | XC5 | 36/0 | 18/0 | 27/0 | 0/0 | 0/0 | 81/0 |
| Fujian | EC1 | 45/0 | 13/0 | 35/0 | 0/0 | 0/0 | 93/0 |
|  | FDDH | 30/86 | 35/54 | 16/22 | 26/0 | 0/0 | 107/0 |
|  | FADB | 47/118 | 131/35 | 70/22 | 0/0 | 0/0 | 248/175 |
|  | ZHDB | 48/91 | 145/13 | 165/77 | 42/0 | 0/0 | 400/0 |
|  | FY6 | 34/91 | 11/19 | 52/43 | 0/0 | 0/0 | 97/153 |
|  | TGY | 0/66 | 24/17 | 102/90 | 0/0 | 0/0 | 126/173 |
|  | HGY | 22/75 | 136/22 | 86/67 | 0/11 | 0/0 | 244/175 |
|  | HJG | 155/128 | 48/12 | 36/34 | 0/0 | 0/0 | 239/174 |
|  | HQ | 73/113 | 40/58 | 29/0 | 0/0 | 0/0 | 142/171 |
|  | MK1 | 146/105 | 64/93 | 0/0 | 0/0 | 0/0 | 210/198 |
|  | MX | 0/102 | 180/23 | 183/55 | 0/0 | 0/0 | 363/180 |
|  | YMX | 120/108 | 44/46 | 44/22 | 44/0 | 0/0 | 252/176 |
|  | FDDB | 0/75 | 0/28 | 0/67 | 0/0 | 0/0 | 0/170 |
| Yunnan | XC11 | 0/0 | 90/0 | 85/0 | 0/0 | 0/0 | 175/0 |
| Zhejiang | LJ43 | 0/28 | 0/72 | 0/20 | 0/63 | 0/0 | 0/0 |
| all |  | 814/2211 | 1389/689 | 2168/1366 | 476/79 | 182/0 | 5029/4345 |

Table S5. The amount of buds, blooming flowers, and withered flowers at different flowering stages

| Tea Flowering Stage | bud | Blooming flower | Withered flower |
| --- | --- | --- | --- |
| Initiation of Flowering Stage | 29780 | 1207 | 1110 |
| Early Peak Flowering Stage | 54035 | 10787 | 5552 |
| Mid Peak Flowering Stage | 84205 | 48255 | 28180 |
| Late Peak Flowering Stage | 12189 | 13043 | 15100 |
| Termination of Flowering Stage | 2200 | 4006 | 8301 |
| all | 182409 | 77298 | 58243 |

Table S6. Comparison of flowering stage between different accessions based on model predictions and manual records.

| Accession | Flowering Stage  (Predicted by model) | Flowering Stage  (manual) |
| --- | --- | --- |
| WN95 | early | early |
| BHZ | early | middle |
| GYQ | early | early |
| AH1 | early | early |
| AH7 | early | early |
| FZ2 | early | early |
| DMB | early | early |
| NZ2 | early | early |
| SMZ | early | early |
| TGY | early | early |
| ZYQ | middle | middle |
| JBH | middle | middle |
| SCZ | middle | middle |
| XC5 | middle | middle |
| EC1 | middle | middle |
| MX | middle | middle |
| FDDH | late | late |
| FADB | late | late |
| ZHDB | late | late |
| FY6 | late | late |
| HGY | late | late |
| HJG | late | late |
| HQ | late | late |
| MK1 | late | late |
| YMX | late | late |
